# Structural studies of phosphorylation-dependent interactions between the V2R receptor and arrestin-2

**DOI:** 10.1038/s41467-021-22731-x

**Published:** 2021-04-22

**Authors:** Qing-Tao He, Peng Xiao, Shen-Ming Huang, Ying-Li Jia, Zhong-Liang Zhu, Jing-Yu Lin, Fan Yang, Xiao-Na Tao, Ru-Jia Zhao, Feng-Yuan Gao, Xiao-Gang Niu, Kun-Hong Xiao, Jiangyun Wang, Changwen Jin, Jin-Peng Sun, Xiao Yu

**Affiliations:** 1 Key Laboratory Experimental Teratology of the Ministry of Education and Department of Biochemistry and Molecular Biology, School of Basic Medical Sciences, Cheeloo college of Medicine, Shandong University, Jinan, Shandong China; 2 Key Laboratory of Molecular Cardiovascular Science, Ministry of Education, Peking University, Beijing, China; 3 School of Life Sciences, University of Science and Technology of China, Hefei, Anhui China; 4 Key Laboratory Experimental Teratology of the Ministry of Education and Department of Physiology, School of Basic Medical Sciences, Cheeloo college of Medicine, Shandong University, Jinan, Shandong China; 5 Beijing Nuclear Magnetic Resonance Center, College of Chemistry and Molecular Engineering, School of Life Sciences, Peking University, Beijing, China; 6 Department of Pharmacology and Chemical Biology, School of Medicine, University of Pittsburgh, Pittsburgh, PA USA; 7 Institute of Biophysics, Chinese Academy of Sciences, Beijing, China; 8 Shenzhen Institute of Transfusion Medicine, Shenzhen Blood Center, Shenzhen, China

**Keywords:** X-ray crystallography, X-ray crystallography

## Abstract

Arrestins recognize different receptor phosphorylation patterns and convert this information to selective arrestin functions to expand the functional diversity of the G protein-coupled receptor (GPCR) superfamilies. However, the principles governing arrestin-phospho-receptor interactions, as well as the contribution of each single phospho-interaction to selective arrestin structural and functional states, are undefined. Here, we determined the crystal structures of arrestin2 in complex with four different phosphopeptides derived from the vasopressin receptor-2 (V2R) C-tail. A comparison of these four crystal structures with previously solved Arrestin2 structures demonstrated that a single phospho-interaction change results in measurable conformational changes at remote sites in the complex. This conformational bias introduced by specific phosphorylation patterns was further inspected by FRET and ^1^H NMR spectrum analysis facilitated via genetic code expansion. Moreover, an interdependent phospho-binding mechanism of phospho-receptor-arrestin interactions between different phospho-interaction sites was unexpectedly revealed. Taken together, our results provide evidence showing that phospho-interaction changes at different arrestin sites can elicit changes in affinity and structural states at remote sites, which correlate with selective arrestin functions.

## Abstract (web summary)

The interaction between a GPCR, such as the vasopressin receptor-2 (V2R), and arrestin depends on the receptors’ phosphorylation pattern. Here authors use FRET and NMR to analyze the phosphorylation patterns of the V2R-arrestin complex and show that phospho-interactions are the key determinants of selective arrestin conformational states and correlated functions.

## Introduction

As the largest transmembrane superfamily, G-protein-coupled receptors (GPCRs) include more than 800 members in the human genome and account for more than 80% of transmembrane signal transduction^1–3^. GPCRs can detect diverse extracellular stimuli and transduce these signals to tens of thousands of different cellular functional outputs, and these signals are mainly transduced via two types of transducers, G proteins and arrestins^1,4–8^. However, the human genome encodes only 16 Gα protein subtypes and four arrestins, numbers that are far lower than the number of numerous receptors with which they interact. Therefore, the principles underlying how each transducer, such as arrestin, recognizes signal inputs from different receptors and translates them to downstream effects are pivotal in governing receptor functions and guiding the design of therapeutics targeting different GPCRs.

The recruitment of arrestin by GPCRs is normally preceded by the phosphorylation of GPCRs by a group of GPCR kinases (GRKs), which generates diverse phosphorylation patterns that determine distinct arrestin functions^1,4–7,9,10^. Several GRK members, such as GRK2 and GRK3, are known to be activated by Gβγ subunits and thus bridge the G protein activation and arrestin pathways^11–13^. Importantly, at least two distinct phospho-GPCR-arrestin complex conformational states have been revealed by cellular and structural studies: (1) the “snug” conformational state, in which arrestin interacts with a receptor through both the seven transmembrane helix (7TM) core and phosphorylated C-tail, or (2) the “hanging” (or “tail”) conformational state, in which arrestin is bound to only the phospho-C-tail of the receptor^4,7,14–17^. In particular, β2-adrenergic-receptor phospho-vasopressin receptor 2-tail (β2AR-ppV2R-tail)-arrestin2-Fab30 complexes in the tail conformational state account for more than 60% of total cryo-EM particles^14^. Moreover, according to recently solved structures of arrestin bound to the phosphorylated vasopressin-2-receptor C-terminus with or without the 7TM core, the “hanging” state could be further divided into two different modes depending on whether the phosphorylated C-terminus engages with the finger loop; these two modes are denoted “hanging (I)” and “hanging (II)” (Supplementary Fig. 1a). Cellular studies have also been performed to differentiate the functional outputs of the “snug” and “tail” conformations. Whereas arrestin in the “snug” conformation is essential for arrestin-mediated G protein desensitization, arrestin in the “tail” conformation can carry out G protein-independent receptor functions, such as ERK activation, ion channel opening, receptor trafficking and routing receptor signaling to SH3 domain-containing molecules^4–6,15–17^ (Supplementary Fig. 1a). Notably, arrestins in the “snug” conformation are governed by the combined efforts of both the 7TM cores and receptor phospho-tail^7,17^. In contrast, arrestins in the “tail” conformation are mainly determined by different phosphobarcodes in the receptor C-tail^14^. Therefore, dissecting the mechanisms governing the selective arrestin conformations encoded by different receptor phospho-patterns and correlating these conformations to specific arrestin functions are key to understanding arrestin-mediated receptor functions.

At least four different receptor-arrestin complex structures in “snug” states, one of rhodopsin complexed with arrestin1 and three of other GPCRs complexed with arrestin2, have been solved using crystallographic or cryo-EM approaches^18–23^. Arrestin1 is specifically expressed in photoreceptor cells and serves as a rapid, sensitive, and dedicated protein that interacts with light-activated rhodopsin, whereas arrestin2 is expressed in many tissues and cells that interact with many GPCR members^16,24^. In addition to cryo-EM, NMR has been used to study receptor-arrestin complex structures in “snug” states. For example, the presence of the 7TM receptor bound to various ligands results in multiple arrestin conformers, as also confirmed by ^1^H NMR using recent TMSiPhe-specific amino-acyl tRNA synthetase technology that allows for site-specific labeling by a trimethyl silyl moiety (DeSipher)^7^. However, no receptor-arrestin complex structure in the “tail” state has yet been solved due to the flexibility of the receptor phospho-C-tail. Through genetic code expansion and ^19^F-NMR measurements, our previous studies proposed a “flute model” to explain the diverse functions induced by different receptor phospho-patterns^6^. Based on this model, we propose that different phospho-binding pockets localized at the arrestin N-terminus serve as individual phospho-sensors and that the binding of phospho-residues to these sensors generates conformational changes at remote functional sites in arrestin, determining the specific arrestin functions. Combinations of these phosphosite-binding patterns are correlated with selective arrestin functions. However, these studies remain preliminary due to the lack of exact information regarding the localized and remote conformational changes caused by binding at each specific phospho-interaction-site, the contributions of binding at each specific phospho-interaction-site to binding affinities, and how binding at each specific phospho-inteaction sites is correlated to diverse signaling molecules downstream of arrestin, such as c-Raf-1, MEK, clathrin, and ERK^25–28^. These questions became particularly interesting when a recently published manuscript indicated that even a single phosphorylation site in V2R could make a decisive contribution to arrestin-mediated signaling^29^.

**Fig. 1:**
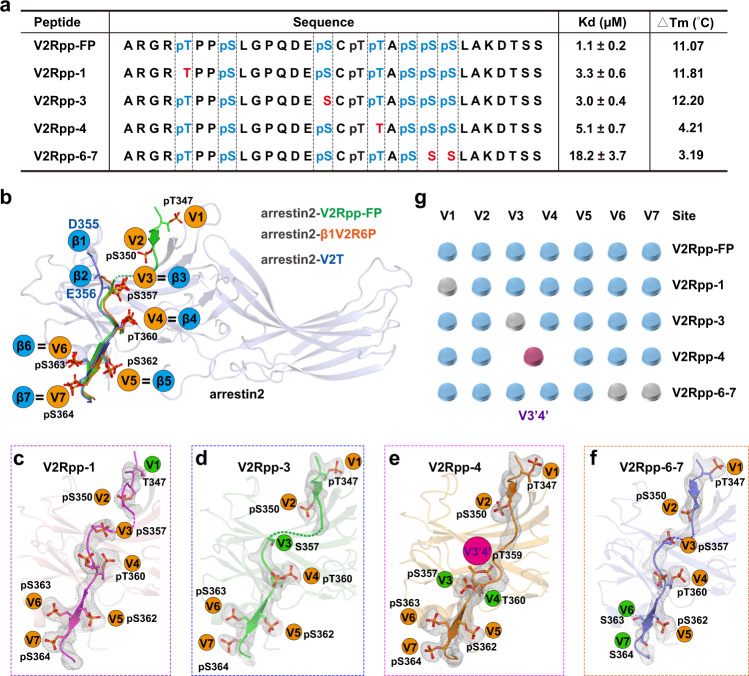
Binding affinities and structures of arrestin2 in complex with four different phosphobarcodes. **a** Sequence representation of V2Rpp-FP, V2Rpp-1, V2Rpp-3, V2Rpp-4, and V2Rpp-6-7. According to a previously published structure, phospho-residues that were expected to bind to arrestin2 are marked blue. The corresponding dissociation constant (Kd value) of each peptide and melting temperature shift (ΔTm value) caused by Fab 30 incubation are shown in the right panels and were determined by Cou-facilitated FRET assay and differential scanning fluorimetry (DSF), respectively. See Supplementary Fig. 2 in the supplementary file. **b** A current model of the phospho-barcoding interaction with arrestin2 according to solved crystal structures or Cryo-EM structures of arrestin2 complexes. The orange balls of V1 to V7 indicate the phospho-binding pockets of arrestin found in the arrestin2-V2Rpp-FP complex (PDB: 4JQI). The blue balls β1–β7 indicate that a phosphate or a negatively charged residue interacts with the specific sites of arrestin, that are determined from the arrestin2-V2T complex (6NI2) and arrestin2-β1V2R6P complex (PDB: 6TKO). **c**–**f** The 2Fo-Fc annealing omits electron density maps of V2Rpp-1, V2Rpp-3, V2Rpp-4, and V2Rpp-6-7. All maps were contoured at 1.0 *σ*. The orange balls indicate that the phosphorylated peptide remains bound to arrestin, as observed in the previously solved arrestin2-V2Rpp-FP complex structure, whereas the green balls indicate loss of the original interaction of the phospho-residue. The purple ball represents the found phospho-binding pocket of arrestin in the arrestin2-V2Rpp-4 complex structure. **g** The specific phospho-binding patterns of arrestin revealed from V2Rpp/arrestin2 complexes. The blue buttons indicate the binding of the selective V2R phospho-peptide in accordance with the predicted phospho-pattern according to the previously solved arrestin2-V2Rpp-FP complex structure. The purple button suggests a discovered phospho-binding pocket of arrestin2 that interacts with V2Rpp-4. Gray buttons indicate that no phosphate interaction was found at the corresponding phospho-binding pockets of arrestin.

In this study, we determined four crystal structures of arrestin-2 in complex with different phosphopeptides derived from the V2 receptor phospho-C-tail, that differ from the fully phosphorylated V2-receptor C-terminal phosphopeptide (V2Rpp-FP) by only one or two phosphates (Fig. 1a) at resolutions ranging from 2.5 to 3.3 Å (Supplementary Table 1). By combining crystallography with BRET and ^1^H NMR (DeSipher) analyses, we were able to correlate these specific “phosphobarcodes” of V2R with conformational changes in arrestin2 and selective downstream signal responses.

## Results

### Phospho-peptide scanning and structure determination

Recent structures of receptor-arrestin2 complexes provided the locations of seven sites that could accommodate phosphates or negatively charged residues. We used the cryo-EM structure of the β1-V2Rpp-arrestin2 complex as a model to name these potential phosphate-binding pockets “β1–β7” (Supplementary Fig. 1b). Alternatively, we used the phospho-binding pockets “V1–V7” to name the mode of V2Rpp-FP binding to arrestin2 in the V2Rpp-FP-arrestin2 (containing the fully phosphorylated vasopressin-2-receptor C-terminal peptide (V2Rpp-FP), which has eight phosphorylated residues) complex structure, as reported previously (Fig. 1b and Supplementary Fig. 1b)^4,6,30^. Notably, five of the phosphate-binding pockets (β3–β7) in the β1-V2Rpp-arrestin2 complex overlapped with the phosphate-binding pockets (V3–V7) defined by the previously solved complex structure of arrestin2-V2Rpp-FP (Fig. 1b and Supplementary Fig. 1b). Whereas these five overlapping phosphate-binding pockets may be common and important in arrestin2 activation induced by many receptors, the “β1-β2” and the “V1–V2” phospho-binding pockets in arrestin2 may serve as important functional switches between the “snug mode” and “hanging mode” in specific cellular contexts (Fig. 1b and Supplementary Fig. 1b).

To understand the distinct arrestin conformations adopted in response to the binding of different receptor phospho-patterns, particularly the contribution of each specific phosphate-binding pocket, we scanned phosphopeptides differing from V2Rpp-FP by only one or two phosphates for their affinity with arrestin and thermostability after complex formation to facilitate structural determination by crystallography (Fig. 1a). We examined the affinities of these phosphopeptides for arrestin using our recently developed FRET method based on genetic expansion^31^. The dissociation constant for the binding of V2Rpp to arrestin2 was approximately 1 μM, and the omission of a phosphate or phosphates from site V1, site V3, site V4 or sites V6–V7 reduced the affinity by approximately 3-fold, 3-fold, 5-fold, and 17-fold, respectively^31^ (Fig. 1a and Supplementary Fig. 2a–2b). To further dissect the changes in arrestin conformation induced by phosphoencoding of different receptors, we set out to crystallize arrestin2 in complex with different phosphopeptides derived from V2Rpp-FP. Because incubation with Fab30, an antibody that is conformationally selective for active arrestins^30^, significantly increased the thermostabilities of the arrestin2/phosphopeptide complexes^32^ (Fig. 1a and Supplementary Fig. 2c–2i), we used Fab30 to facilitate crystallization (Supplementary Fig. 3). Arrestin2 in complex with V2Rpp-1, V2Rpp-3, V2Rpp-4, and V2Rpp-6-7, which lack the original phosphate that binds V1, V3, V4, and V6/V7, respectively, was successfully crystallized, and the complex structures were determined at resolutions of 3.2 Å, 2.5 Å, 2.5 Å, and 3.3 Å, respectively (Supplementary Table 1). The arrestin2-V2Rpp-1, arrestin2-V2Rpp-3, arrestin2-V2Rpp-4 and arrestin2-V2Rpp-6-7 structures were finally refined, giving R_free_ values of 0.288, 0.285, 0.281, and 0.290, respectively (Supplementary Table 1).

### Distinct phospho-binding patterns in active arrestin structures

In all four solved arrestin2-phosphopeptide complex structures, the electron density for the bound phosphopeptide was clearly defined, and the phosphates in the phosphopeptides were unambiguously assigned (Fig. 1c–f). Consistent with our hypothesis, specific phospho-binding was absent at the specific phosphorylation deletion site in the arrestin2-V2Rpp-1, arrestin2-V2Rpp-3, and arrestin2-V2Rpp-6-7 complex structures, whereas all other phospho-interactions in the previously solved arrestin2-V2Rpp-FP^30^ structure were preserved in these three complex structures (Fig. 1c, d, f, g). However, the arrestin2-V2Rpp-4 complex structure unexpectedly caused rearrangement of both the 3rd (pS357) and 4th (pT359) phospho-residues. Consequently, V2Rpp-4 did not show binding within both the V3 and V4 pockets to arrestin2, and pT359 formed a different phospho-interaction, which was named the V3’4’ site (Fig. 1e, g). The interaction mode by V2Rpp-4 was not predicted and has not been previously observed, which not only suggests the plasticity of the arrestin phospho-binding cavity, but also indicates that the binding mode of the receptor phospho-C-tail to arrestin could not be simply derived from the primary sequence of a receptor-phospho-C-tail.

### Arrestin in active conformations

**Fig. 2:**
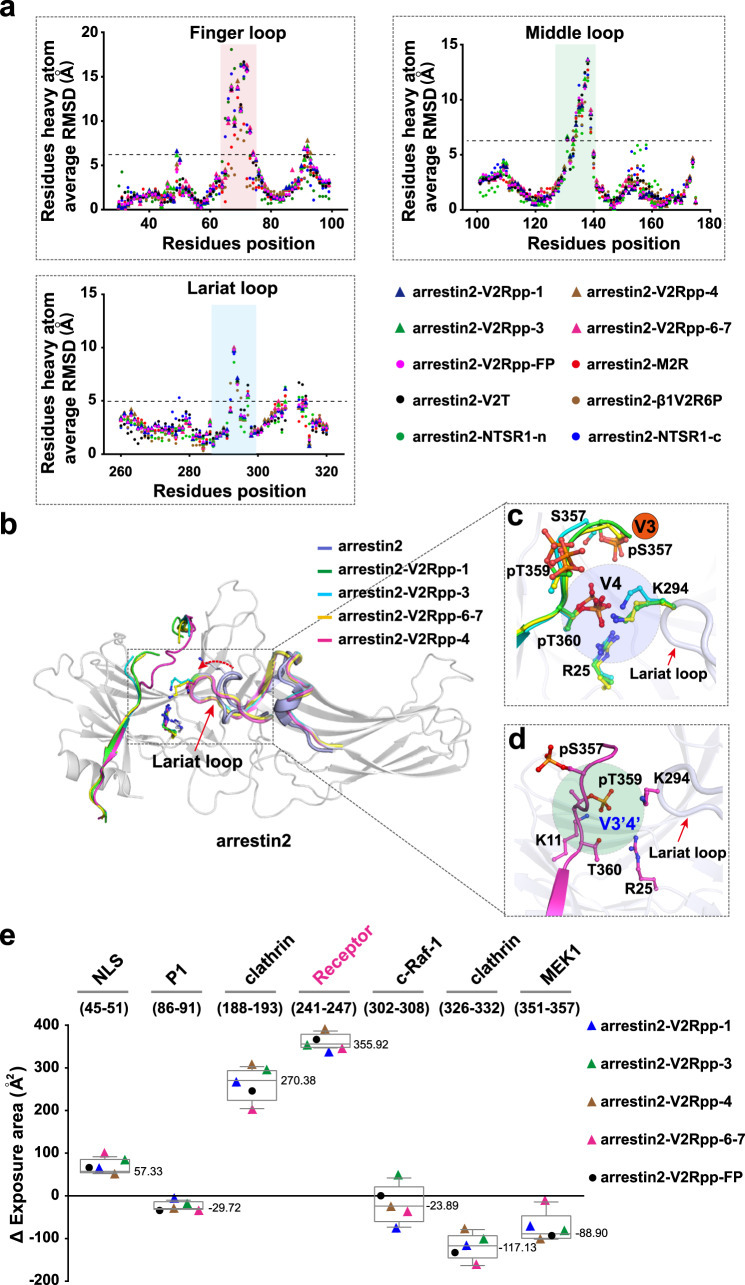
Phosphopeptides induce arrestin to form an active conformation. **a** Plots of the distance root mean square deviations (RMSDs) for individual residues between inactive arrestin2 and the V2Rpp-FP/1/3/4/6-7-arrestin2 complexes. The three loops that exhibit major conformational changes in all complexes are highlighted: finger loop, light red; middle loop, light green; and lariat loop, light blue. Arrestin2-V2Rpp-FP, PDB: 4JQI; arrestin2-NTSR1-c, PDB: 6PWC; arrestin2-NTSR1-n, PDB: 6UP7; arrestin2-M2R, PDB: 6U1N; arrestin2-β1AR6P, PDB: 6TKO; arrestin2-V2T, PDB:6NI2. **b** Similar conformational distortions of the Lariat loop in the V2Rpp-1/3/4/67-arrestin2 complex. **c** pT360 of V2Rpp-1/3/67 maintained H-bond interactions with residues K294 and R25, similar to V2Rpp-FP. **d** A different binding mode of V2Rpp-4. V2Rpp-4 lost both the V3 and V4 site interactions. Instead, it formed a phospho-interaction via the identified V3′4′ site. **e** The solvent accessibilities of several functional regions in arrestin2 in five different phosphopeptides-arrestin2 complex structures (Black circle: arrestin2-V2Rpp-FP, PDB: 4JQI; Blue triangle: arrestin2-V2Rpp-1; green triangle: arrestin2-V2Rpp-3; brown triangle: arrestin2-V2Rpp-4; pink triangle: arrestin2-V2Rpp-6-7). Data are reported as box and whiskers plot, the whiskers show the maximum and the minimum; the box indicates the first and third quartiles; and the line in the box is the median value. The median values are reported next to the boxplot. The different color triangles and circle represent the five different phosphopeptides-arrestin2 complex structures’ averaged for the solvent accessibilities in corresponding regions. Source data are provided in the Source Data file.

Arrestin activation is characterized by interdomain twisting, repositioning of the three loops (finger loop, middle loop, and lariat loop), and breaking of the polar core^18–21,30,33^. Compared with inactive arrestin, all of our solved arrestin structures bound to the V2R phosphopeptides exhibited substantial structural rearrangements at the three loops (Fig. 2a), and this effect was accompanied by a 16° to 21.6° rotation of the C-lobe relative to the N-lobe (Supplementary Fig. 4).

The polar core of inactive arrestin2 is maintained by three negatively charged residues and two positively charged residues, which are D26, D290, D297, R169, and R393. Disruption of the polar core is likely mediated by at least two processes: displacement of the arrestin C-terminal residue R393 and twisting of the lariat loop. In all four structures, the C-terminal tail bound to the phospho-binding cavity was replaced by the V2R-phosphopeptides, and this replacement was accompanied by distortion of the lariat loop (Fig. 2b). Interestingly, the twisting of the lariat loop allowed H-bond engagement of residue K294 with pT360 in the arrestin2-V2Rpp-1, arrestin2-V2Rpp-3 and arrestin2-V2Rpp-6-7 complex structures, which was hypothesized to be the key interaction anchoring the active lariat loop conformation (Fig. 2c), but this specific interaction with K294 and the interaction of pT360 with R25 were lost in the arrestin2-V2Rpp-4 structure despite a similar twisting of the lariat loop (Fig. 2d). Combined with the fact that the contribution of each phosphate to the overall binding affinity is quite small, ranging from 3-fold to 5-fold, these observations suggested that the observed disruption of the polar core of arrestin is not due to the binding of a key phosphorylation site but is determined by displacement of the arrestin C-terminus and the presence of excess negative charges in the phospho-binding cavity of arrestin.

Conformational changes in at least six functionally related arrestin regions were noted in these solved structures of arrestin activated by different phospho-patterns (Fig. 2e, Supplementary Fig. 5, and Supplementary Table 2). The solvent accessibilities of these regions in the structures of arrestin activated with different phospho-patterns were then compared with those of both the inactive arrestin2 and arrestin2-V2Rpp-FP complex structures, which revealed that these structures exhibited more solvent exposure at a potential receptor-binding site at similar levels (Fig. 2e). Notably, the solvent exposure of interfaces involved in the scaffolding of downstream effectors of receptors, such as c-Raf-1, MEK1, or SRC (P1), changed in different ways in response to engagement with different phospho-patterns (Fig. 2e). These observations elicited great interest in investigating the detailed conformational changes induced by each specific phospho-pattern and characterizing their structure-function relationships.

### Binding of the V1 site of arrestin is linked to conformational changes at the c-Raf-1-binding site

**Fig. 3:**
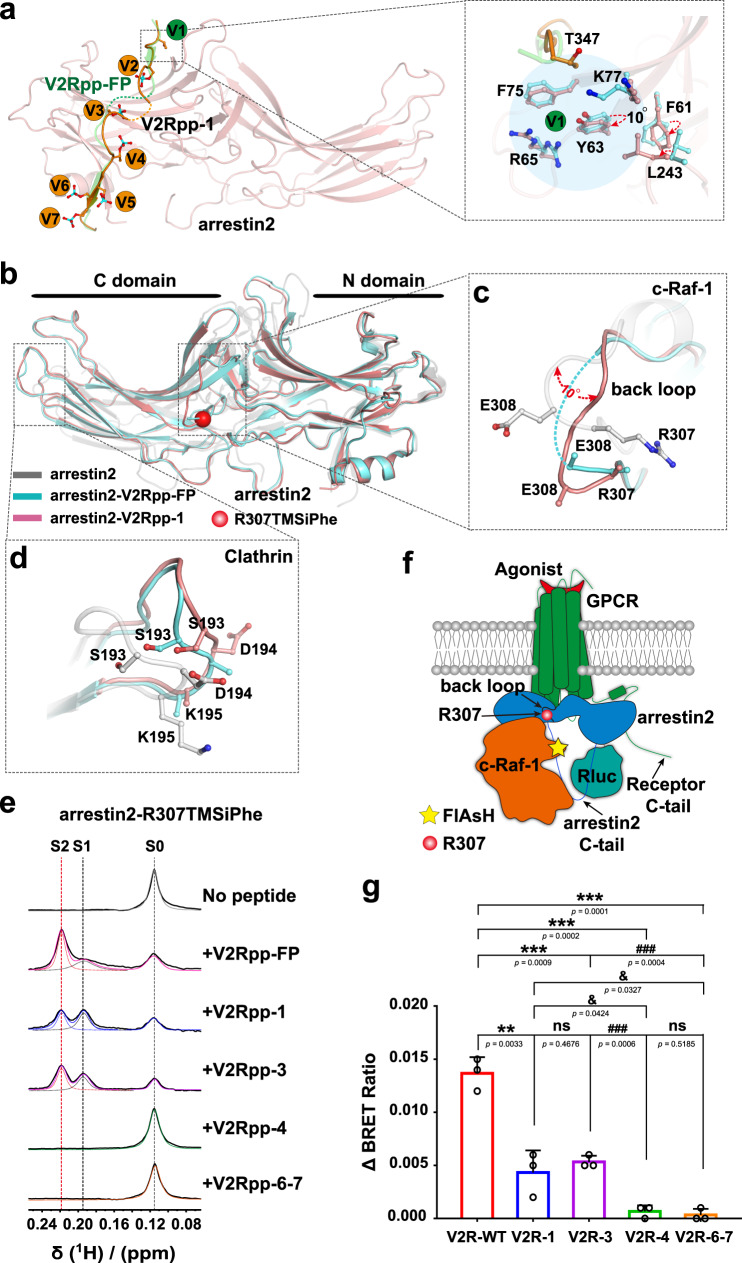
Structure of the arrestin2-V2Rpp-1 complex and correlation of phospho-binding at the V1 site with the conformational change related to the c-Raf-1 interaction. **a** Structural comparisons of residues near the V1 site in the structure of the arrestin2-V2Rpp-1 complex (salmon) and arrestin2-V2Rpp-FP (PDB: 4JQI, light cyan). Residues F61, Y63, and L243 underwent obvious structural rearrangement. The orange balls indicate that the phosphorylated peptide remains bound to arrestin, as observed in the previously solved arrestin2-V2Rpp-FP complex structure, whereas the green balls indicate loss of the original interaction of the phospho-residue. **b** Overall structural comparisons of the arrestin2-V2Rpp-1 complex (salmon), arrestin2-V2Rpp-FP complex (PDB: 4JQI, light cyan) and inactive arrestin2 (PDB:1G4M, gray). R307TMSiPhe was marked as red ball. **c** Comparisons of conformational changes at the back loop in the arrestin2-V2Rpp-1 complex with the inactive arrestin2 and arrestin2-V2Rpp-FP complex. The back loop experienced conformational rearrangement by approximately 10° rotation in the arrestin2-V2Rpp-1 complex compared to inactive arrestin2. **d** The loop at the region from S193 to K195 underwent significant structural rearrangement in arrestin2-V2Rpp-1 compared to that in the arrestin2-V2Rpp-FP complex structure. **e** 1D ^1^H NMR spectra of arrestin2-R307TMSiPhe in response to incubation with different phosphopeptides or control vehicles. The corresponding NMR shifts at 0.116, 0.195, and 0.219 ppm are designated S0, S1, and S2, respectively. **f** Schematic representation of the experimental design used to monitor vasopressin-induced c-Raf-1 recruitment to arrestin2 downstream of V2R. The interaction between arrestin2 and c-Raf-1 was measured by the FlAsH-BRET method. The red ball: R307 site in arrestin2; Yellow pentagram: the position of FlAsH labeled in c-Raf-1. **g** Vasopressin-induced recruitment of c-Raf-1 to arrestin2 in HEK293 cells overexpressing wild-type V2R or different mutants. Data are expressed as relative values of ΔBRET ratio with the mean ± SEM of three independent experiments (*n* = 3). Statistical differences were determined by two-sided one-way ANOVA with Tukey’s test. * on behalf of differences between V2R-WT and mutants; *P < 0.05; **P < 0.01; ***P < 0.001; & on behalf of differences between V2R-1 and V2Rpp-4, V2Rpp-6-7 mutants; &*P* < 0.05; &&*P* < 0.01; &&&*P* < 0.001; # on behalf of differences between V2R-3 and V2Rpp-4, V2Rpp-6-7 mutants; #*P* < 0.05; ##*P* < 0.01; ###*P* < 0.001; ns no significant difference. Equal surface expression of all V2R constructs was achieved by adjusting the amounts of transfected plasmids and monitored by ELISA. Source data are provided in the Source. Data file.

In the complex structure of arrestin bound to V2Rpp-FP, pT347 formed hydrogen bonds or charge-charge interactions with Y63, R65, and K77 and an anion-π interaction with F75 (Supplementary Table 3). These residues were located at the finger loop and underwent substantial structural rearrangements in response to phosphopeptide interactions. The binding of either V2Rpp-FP in the previously solved arrestin2-V2Rpp-FP complex structure or V2Rpp-1 in our solved arrestin2-V2Rpp-1 structure disrupted the ionic locks, that formed between R62-E66 and D69-R76 in the inactive arrestin2 structure, which caused a nearly 90° twisting of the finger loop (Fig. 3a and Supplementary Fig. 6). Compared with the V2Rpp-FP structure, the absence of the phospho-interaction at the V1 site in the arrestin2-V2Rpp-1 complex structure resulted a lack of interactions between the peptide and Y63, R65, and F75. The movement of K77 away from T347 in the arrestin2-V2Rpp-1 complex structure resulted in loss of the H-bond that formed between K77 and pT347 in the arrestin2-V2Rpp-FP complex structure (Fig. 3a, Supplementary Fig. 7a, and Supplementary Table 3). Loosing these interactions caused approximately 10° tilting of the phenyl ring of Y63 and repositioning of the side chain of R65 and therefore potentially promoted structural rearrangement of the packing between L243 and F61, which might establish a path for the propagation of conformational changes toward remote functionally relevant sites (Fig. 3a).

Importantly, RMSD profiling between the complex structures of arrestin2-V2Rpp-1 and arrestin2-V2Rpp-FP revealed that eight residues exhibit changes in the main chain Cα coordinates between 1.5 and 4 Å, and these are concentrated at the β4–β5 and β5–β6 turns and three functional sites, including the nuclear localization sequence (NLS) and the c-Raf-1-binding and clathrin-binding sites (Supplementary Figs. 8a, 8b and 9a). Remarkably, the back loop in the arrestin2-V2Rpp-1 complex structure experienced obvious conformational rearrangement, as characterized by a rotation of approximately 10°, compared with the results obtained for inactive arrestin2. The electron density of the back loop of arrestin2-FP was not clear, which suggested that the conformation of this specific structural motif in the arrestin2-FP structure was more flexible than that in arrestin2-V2Rpp-1 (Fig. 3b, c and Supplementary Fig. 10a). The β-XI-β-XII loop (encompassing S193 to K195), which is one of the potential clathrin-binding sites, also underwent significant structural rearrangement in the arrestin2-V2Rpp-1 complex compared with the results obtained for the arrestin2-V2Rpp-FP complex structure (Fig. 3b, d and Supplementary Fig. 10b).

To confirm the conformational changes observed in the crystal structure, we used our recently developed ^1^H NMR of a trimethylsilyl moiety method (DeSipher method) to characterize the V2Rpp-1-induced arrestin conformational changes at the β4–β5 turn and c-Raf-1-binding sites^7^, and compared these with the signals obtained with and without stimulation with V2Rpp-FP (Supplementary Fig. 11a–e). Previous studies have shown that R307 in the back loop of arrestin2 is involved in the interaction with c-Raf-1. The R307A mutation in arrestin2 significantly reduced the interaction between arrestin2 and c-Raf-1^25^. The high flexibility of the back loop limits the precise structural definition of the c-Raf-1-binding site in this region. We therefore incorporated TMSiPhe at the R307 position to monitor the conformational change in this specific c-Raf-1-binding region of arrestin. All DeSipher probes used in the current study showed efficient TMSiPhe incorporation without a change in functional integrity (Supplementary Fig. 12 and Supplementary Fig. 18). The ^1^H NMR spectrum of arrestin2 R307TMSiPhe in the inactive state contained one main peak at 0.116 ppm in the range from 0 to 0.24 ppm, which was designated S0 (Fig. 3e, Supplementary Fig. 11d, e, and Supplementary Table 4). Upon stimulation with 100 μM V2Rpp-FP, the peak volume of S0 significantly decreased, and this decrease was accompanied by the appearance of two peaks at 0.195 ppm and 0.219 ppm, which were designated S1 and S2, respectively (Fig. 3e, Supplementary Fig. 11d, e, and Supplementary Table 4). Truncation of the arrestin2 C-terminus is known to cause arrestin2 to adopt an intermediate state between the inactive and active arrestin conformations^30,34,35^. We therefore measured the ^1^H NMR spectrum of arrestin2 1–382 R307TMSiPhe, which was purified to homogeneity (Supplementary Fig. 13a). The arrestin2 1–382 truncation resulted in loss of the autoinhibition of its C-tail, which exhibits intramolecular interactions with several phospho-binding pockets. In the absence of phosphopeptide binding, arrestin2 1-382-R307TMSiPhe presented a primary NMR signal at 0.122 ppm (designated S0′) (Supplementary Fig. 13b, c and Supplementary Table 5). Incubation with V2Rpp-FP resulted in the disappearance of the S0′ peak and emergence of the S2 peak and a secondary peak at 0.184 ppm (designated S1′ here) (Supplementary Fig. 13c and Supplementary Table 5). The common S2 peak in the NMR spectra of both arrestin2 R307TMSiPhe and arrestin2 1-382 R307TMSiPhe might indicate that arrestin2 has adopted a “full activation” conformational state in response to the V2Rpp-FP interaction, whereas the S0′ and S1 states might reflect a conformational intermediate between inactive (S0) arrestin2 and active (S2) arrestin2 bound to V2Rpp-FP. Collectively, these NMR spectra reflected conformational changes of the R307 site in the transition of arrestin from the inactivate conformation to an active state.

Similar to the results obtained with V2Rpp-FP, the application of V2Rpp-1 to arrestin2 caused a reduction in the S0 peak, and this reduction was accompanied by the appearance of S1 and S2 peaks (Fig. 3e and Supplementary Table 4). However, the S2 peak in the arrestin2-V2Rpp-1 complex was markedly smaller than that in the arrestin2-V2Rpp-FP structure, and the S3 peak was relatively larger (Fig. 3e and Supplementary Table 4). The ^1^H-NMR spectrum was consistent with the observations from the crystal structures, which indicates that the presence of phospho-binding at the V1 site could induce different conformations of the back loop, which is a previously characterized c-Raf-1-binding site^25^.

We subsequently examined the effect of the V2R-T347A (V2Rpp-1 site) mutation on the recruitment of c-Raf-1 to arrestin in response to the stimulation of HEK293 cells overexpressing wild-type or mutant V2R with vasopressin. Wild-type V2R and all corresponding mutants were expressed at approximately equal levels by adjusting the amounts of the plasmids used for transfection (Supplementary Fig. 14a, b), and an arrestin2 recruitment assay was performed. The EC_50_ values of the vasopressin-induced V2R-arrestin2 interactions for different V2R mutants and the wild-type V2R were similar; however, the Emax of the V2R-S357A mutant (V2Rpp-3 site) was decreased to 60% compared to that of wild-type V2R, and the Emax values of V2R-T360A (V2Rpp-4 site) and the V2R-S363A/S364A (V2Rpp-6-7 site) mutants were decreased to approximately 30% compared to that of wild-type V2R, which suggested that less arrestin2 was recruited to the V2R mutant than to the wild-type V2R over a given time (Supplementary Fig. 14c, d). We then attached Rluc to the N-terminus of arrestin2 and incorporated the FlAsH motif between R125-L126 in c-Raf-1 to monitor the interactions between arrestin2 and c-Raf-1 (Fig. 3f, g). The c-Raf-1-FlAsH probe was able to functionally couple with active arrestin2, as supported by coimmunoprecipitation (co-IP) assays (Supplementary Figs. 15 and  19). Importantly, significantly less c-Raf-1 was recruited to arrestin by the T347A mutant of the V2 receptor than by the wild-type V2 receptor (Fig. 3g), which is consistent with the decreased volume of the S2 peak in response to V2Rpp-1 stimulation compared with that in response to V2Rpp-FP stimulation obtained by NMR. These results indicated that the binding of phosphosite V1 might be correlated with the recruitment of c-Raf-1 kinase to active V2R-arrestin complexes.

### Structure and function of the V2Rpp-3/Arrestin2 complex

**Fig. 4:**
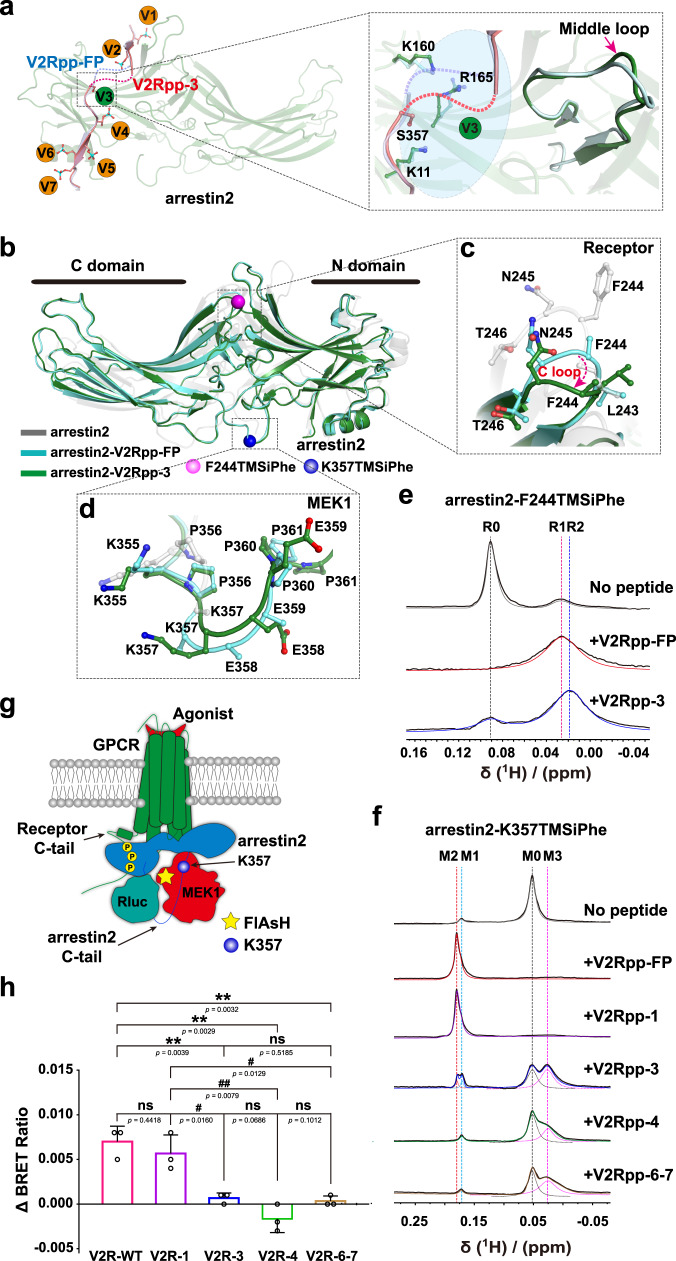
Structure of the arrestin2-V2Rpp-3 complex and the conformational change related to MEK1 interaction. **a** The loss of pS357 of V2Rpp-3 caused rotamer alterations in the side chains of R165, K138, and K160 of the V3 binding pocket. The orange balls indicate that the phosphorylated peptide remains bound to arrestin, as observed in the previously solved arrestin2-V2Rpp-FP complex structure, whereas the green balls indicate loss of the original interaction of the phospho-residue. **b** Overall structural comparisons of the arrestin2-V2Rpp-3 complex (forest) with the arrestin2-V2Rpp-FP complex (PDB: 4JQI, light cyan) and inactive arrestin2 (PDB:1G4M, gray). F244TMSiPhe and K357TMSiPhe were marked as pink ball and blue ball, respectively. **c** Conformational changes occurred at the C loop (F243-T246), which is known to be involved in receptor binding. **d** Conformational changes in the region involved in the MEK1 interaction (K355-P361). **e** 1D ^1^HNMR spectra of arrestin2-F244TMSiPhe in response to incubation with different phosphopeptides. Corresponding NMR shifts at 0.091 ppm, 0.026 ppm, and 0.019 ppm were assigned to the R0, R1, and R2 states, respectively. **f** 1D ^1^HNMR spectra of arrestin2-K357TMSiPhe in response to incubation with different phosphopeptides. Corresponding NMR shifts at 0.055 ppm, 0.170 ppm, 0.180 ppm, and 0.026 ppm were designated M0, M1, M2, and M3, respectively. **g** Schematic representation of the experimental design used to monitor vasopressin-induced MEK1 recruitment to arrestin2 downstream of V2R by FlAsH-BRET between arrestin2-Rluc and MEK1-FlAsH. The blue ball: K357 site in arrestin2; Yellow pentagram: the position of FlAsH labeled in MEK1. The yellow circles: phosphorylation. **h** Vasopressin-induced recruitment of MEK1 to arrestin2 in HEK293 cells overexpressing wild-type V2R or different mutants. Data are expressed as ΔBRET ratio relative values and represent the mean ± SEM of three independent experiments (*n* = 3). Statistical differences were determined by two-sided one-way ANOVA with Tukey’s test. * on behalf of differences between V2R-WT and mutants; *P < 0.05; **P < 0.01; ***P < 0.001; # on behalf of differences between V2R-1 and V2Rpp-3, V2Rpp-4, V2Rpp-6-7 mutants; #*P* < 0.05; ##*P* < 0.01; ###*P* < 0.001; ns no significant difference. Source data are provided in the Source Data file.

pS357 engaged via both polar and charge-charge interactions with K11 in the N-terminal arm, R165 in the β-strand X, K138 in the middle loop and K160 in the phospho-sensor region of the arrestin2-V2Rpp-FP complex structure (Fig. 4a and Supplementary Table 6). Compared with the arrestin2-V2Rpp-FP complex structure, the loss of the pS357-mediated interactions in the arrestin2-V2Rpp-3 complex structure caused rotamer alterations in the side chains of R165 and K160 (Fig. 4a and Supplementary Fig. 7b), and a slight shift in the main chain of the middle loop (Fig. 4a).

An examination of the RMSD between the arrestin2-V2Rpp-FP and arrestin2-V2Rpp-3 complex structures identified 14 residues, whose Cα atoms differ in position by 1.5–4 Å, including several functional sites related to MEK1 or receptor interactions (Supplementary Figs. 8c, d and 9b). Compared with those in the arrestin2-V2Rpp-FP complex structure, the F244-N245 sites were farther away from the phosphopeptide-binding cavity, and K357-E358 exhibited higher solvent exposure (Fig. 4b–d and Supplementary Fig. 10c, d). Importantly, F244-N245 were hypothesized to participate in receptor core interactions, whereas K357-E358 are located in the C-tail region (350–393) and participate in MEK1 interactions^26^ (Figs. 2e and 4c, d). We then investigated the conformational rearrangements of arrestin2 at these corresponding positions by ^1^H NMR (Fig. 4e, f and Supplementary Tables 7–8). The ^1^H NMR spectrum of arrestin2-F244TMSiPhe in the inactive state showed a main peak at 0.091 ppm, which was designated R0, and a small peak at 0.026 ppm (R1). The addition of V2Rpp-FP or V2Rpp-3 caused a significant decrease in the volume of the R0 peak. In addition, incubation with V2Rpp-FP increased the volume of R1, and the administration of V2Rpp-3 induced the appearance of a peak at 0.019 ppm (R2) (Fig. 4e and Supplementary Table 7). The NMR measurements thus confirmed the conformational heterogeneity at the arrestin2-F244 site in response to stimulation with different phosphobarcodes. Importantly, a recently published study indicated that the loss of potential V3/β3 phospho-binding by the S357A mutant of the V2 receptor significantly decreased its arrestin recruitment ability^29^. Because the F244 site is known to be involved in receptor interactions, the different conformational states of F244 in arrestin2 observed in our NMR experiments might thus contribute to the different receptor/arrestin association capabilities caused by the absence of V3/β3 phospho-binding.

We then inspected the ^1^H NMR spectrum using the K357TMSiPhe probe. A primary NMR signal appeared at 0.055 ppm (designated M0), and an additional small peak located at 0.17 ppm was designated M1. The incubation of V2Rpp-FP or V2Rpp-1 caused similar changes in the ^1^H-NMR spectrum, which included elimination of the M0 peak and the formation of a peak at 0.18 ppm (M2) (Fig. 4f and Supplementary Table 8). Interestingly, arrestin2 1-382 K357TMSiPhe exhibited a main peak at 0.110 ppm (designated M0′) in the ^1^H-NMR spectra and a minor M2 peak. This result further supported the finding that M2 likely represents the active state, because arrestin2 1-382 was assumed to be more active than full-length arrestin2 in the basal state. In response to engagement with V2Rpp-FP, the ^1^H-NMR spectrum of arrestin2 1–382 K357TMSiPhe showed an identical NMR signal to that of arrestin2 K357TMSiPhe (Supplementary Fig. 13d and Supplementary Table 9). Collectively, these results indicated that the M0 state resembled an inactive state at the K357 site of arrestin2, M2 indicated an active state, and M0′ was likely an intermediate state. Importantly, a different spectrum pattern of the K357TMSiPhe probe was observed after the incubation of arrestin2 with V2Rpp-3, V2Rpp-4, and V2Rpp-67, and the changes included a decrease in the M0 peak and the appearance of the M3 peak at 0.026 ppm (Fig. 4f and Supplementary Table 8).

Previous studies have shown that the C-tail of arrestin2 (including the K357 site) is involved in the MEK1 interaction^26^. We therefore designed a BRET experiment to examine the effects of changes in the V2 receptor phospho-binding pattern on the interactions between arrestin2 and MEK1 downstream of V2 receptor activation. A FlAsH motif was incorporated between the T238 and H239 sites of MEK1. The MEK-FlAsH probe was found to functionally couple with active arrestin2 (Supplementary Figs. 15 and 19). Vasopressin significantly increased the recruitment of MEK1 to arrestin2 in both the wild-type V2 receptor and the V2 receptor T347A mutant corresponding to V2Rpp-1. However, the elimination of the potential phosphate interactions at V3, V4, and V6–V7 by the S357A, T360A, and S363A-S364A mutations of V2R, respectively, almost abolished the MEK1-arrestin2 association in response to vasopressin stimulation (Fig. 4g, h). Therefore, the M2 state might reflect a conformational state of the arrestin2 K357 site that favors MEK1 interaction, whereas the M0 and M3 peaks might correlate a conformational state of the arrestin2 K357 site that is not conducive for MEK1 interaction. It is worth-noting that the M3 state located at the higher field compared with the M0 state, whereas M1 and M2 located at the lower field. This finding might indicate that K357 may move away from the negatively charged residues E358 and E359 or become more buried in the M2 state, thus enabling a more feasible interaction with MEK1 than that with the M0 and M3 states.

### Structure and function of V2Rpp-6-7/Arrestin2 complex

**Fig. 5:**
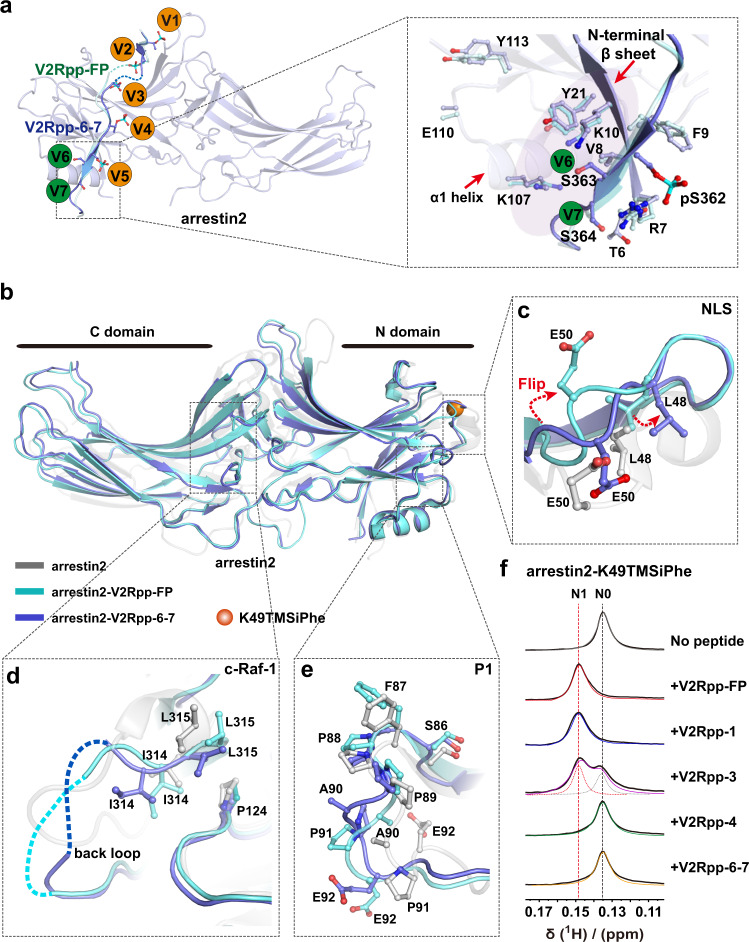
Structure of the arrestin2-V2Rpp-6-7 complex. **a** Structural alterations around sites V6 and V7 in the arrestin2-V2Rpp-6-7 complex structure (blue). Compared to the arrestin2-V2Rpp-FP complex, loss of pS363 and pS364 in V2Rpp-6-7 caused rotations of the side chains of direct phospho-contacting residues, such as K10, Y21, and K107, as well as structural reorganization at the N-terminal β sheet and α1 helix. The orange balls indicate that the phosphorylated peptide remains bound to arrestin, the green balls indicate loss of the original interaction of the phospho-residue. **b** Overall structural comparisons of arrestin2-V2Rpp-6-7 (slate) with the arrestin2-V2Rpp-FP complex (PDB: 4JQI, light cyan) and inactive arrestin2 (PDB:1G4M, gray). K49TMSiPhe was marked as orange ball. **c** The side chains of L48 and E50 at the NLS site in the arrestin2-V2Rpp-6-7 complex flipped towards the solvent compared with the same residues in the arrestin2-V2Rpp-FP complex. **d** Structural comparisons of the back loop in V2Rpp-6-7 bound arrestin2. **e** Structural rearrangement at the P1 region in the arrestin2-V2Rpp-6-7 complex. **f** 1D ^1^H NMR spectra of arrestin2-K49TMSiPhe incubated with different phosphopeptides.

Elimination of the last two phosphorylation sites at the S363 and S364 positions in V2Rpp-6-7 caused an approximately 17-fold decrease in the phosphopeptide-binding affinity of V2Rpp-FP (Fig. 1a), which demonstrated the importance of these specific interactions. The pS363 and pS364 residues in arrestin2-V2Rpp-FP formed hydrogen bonds and charge–charge interactions with K10, Y21, and K107 (Supplementary Table 10). The binding of V2Rpp-FP and V2Rpp-6-7 caused an approximately 3-Å shift in the N-terminal α-helix and reorganization of the β sheet VII-α1 bulge due to replacement of the arrestin C-tail by the bound phosphopeptides (Supplementary Fig. 16). Compared with the arrestin2-V2Rpp-FP structure, the loss of the two phosphates at S363 and S364 in V2Rpp-67 not only caused side chain rotamer shifts at direct contacting residues, such as K10, Y21, and K107, but also caused structural rearrangements at the N-terminal β sheet (T6-K10) and residues located at the α1 helix, such as E110 and Y113. These structural alterations could propagate to cause distal conformational changes in V2R-pp-bound arrestin2 (Fig. 5a and Supplementary Fig. 7c).

Examination of the RMSD between the arrestin2-V2Rpp-FP and arrestin2-V2Rpp-6-7 complex structures uncovered 12 residues that exhibited Cα differences between 1.5 and 4 Å, and these residues included the NLS site, P1 region (SRC interaction site), clathrin region and c-Raf-1 region (Supplementary Figs. 8e, 8f and 9c). At the NLS site, the side chains of L48 and E50 in the arrestin2-V2Rpp-6, 7 complex were flipped toward the solvent compared with those of the same residues in the arrestin2-V2Rpp-FP complex structure (Fig. 5b, c and Supplementary Fig. 10e). In the back loop, which is the c-Raf-1-interacting region, the residue L315 was packed more closely to P124, and I314 became more solvent exposed (Fig. 5b, d and Supplementary Fig. 10f). Moreover, significant structural rearrangement was detected in the P1 region (Fig. 5b, e and Supplementary Fig. 10g).

We therefore inspected the conformational changes at the NLS site of arrestin2 using DeSipher technology. The ^1^H NMR spectrum of arrestin2-K49TMSiPhe in the inactive state exhibited a main peak at 0.135 ppm, which was designated N0. The appearance of a peak appeared at 0.149 ppm (N1 state) accompanied by elimination of the N0 state was observed in response to either V2Rpp-FP or V2Rpp-1 stimulation. Abolition of the phosphate-binding site of V3 partially transferred the N0 state of arrestin2 to the N1 state, and elimination of phosphate binding at the V6 and V7 sites in V2Rpp-6-7 or at the V4 site removed the abilities of these peptides to induce the N1 conformational state of arrestin2 (Fig. 5f and Supplementary Table 11). In conclusion, the phosphorylation of S363 and S364 plays an important role in regulating the NLS conformation.

We subsequently examined the effect of V2Rpp-6-7 stimulation on the ^1^H NMR spectrum of R307TMSiPhe of arrestin2, which serves as a conformational sensor for the c-Raf-1 interaction. Consistent with the results from the crystallographic analysis, the elimination of phosphate binding at the V6 and V7 sites in V2Rpp-6-7 produced a ^1^H-NMR spectrum that was quite different from that of V2Rpp-FP, but very similar to that obtained with the control vehicle treatment (Fig. 3e). The NMR spectrum pattern was consistent with the BRET experimental results because the vasopressin-induced interaction between c-Raf-1 and arrestin2 was abolished by the S363A-S364A mutations in V2R (Fig. 3g). A recent study suggested that phospho-clustering at the S363-S364 sites of the V2R was responsible for stimulating the recruitment of effectors downstream of arrestins^29^. Our results therefore provide direct evidence supporting this hypothesis regarding the structure-function relationship of phospho-binding at the V6 and V7 sites.

### The different phospho-binding mode of arrestin

**Fig. 6:**
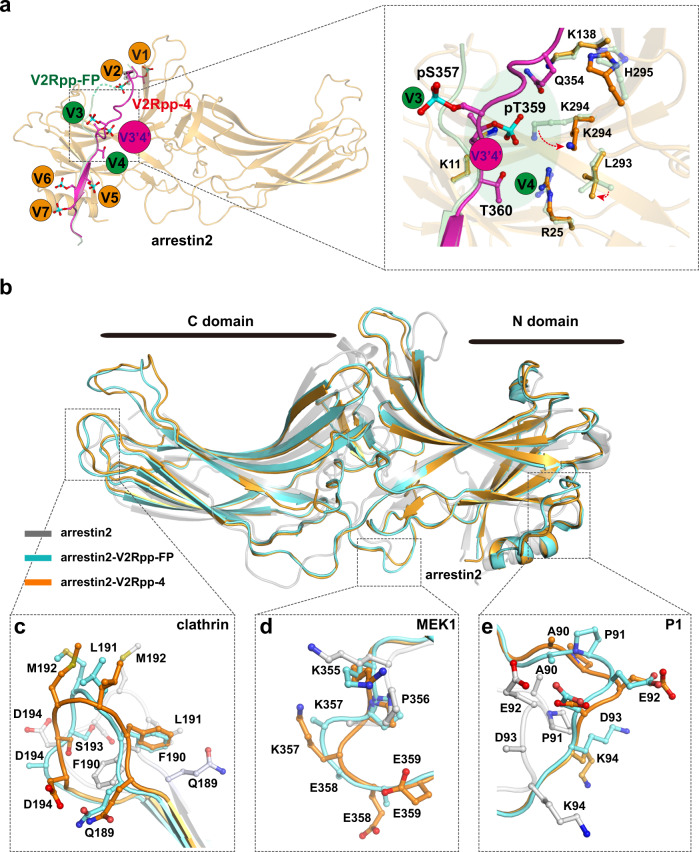
An alternative phospho-binding mode in the arrestin2-V2Rpp-4 complex. **a** pT359 bound at the V3′4′ site in the arrestin2 complex-V2Rpp-4 complex structure. Loss of pT360 in V2Rpp-4 altered the conformation of the phospho-segment ranging from P353 to T360, leading to the loss of the phospho-interaction at both the V4 site and V3 site and the transposition of pT359 to form hydrogen bond or charge-charge interactions with K11, K294, or R25 of arrestin2, respectively. This identified phospho-interaction site was designated the V3′4′ site. The orange balls indicate that the phosphorylated peptide remains bound to arrestin, the green balls indicate loss of the original interaction of the phospho-residue. The purple ball represents the found phospho-binding pocket of arrestin in the arrestin2-V2Rpp-4 complex structure. **b** Overall structural comparisons of arrestin2-V2Rpp-4 (orange) with the arrestin2-V2Rpp-FP complex (PDB: 4JQI, light cyan) and inactive arrestin2 (PDB:1G4M, gray). **c** V2Rpp-4 stabilized the clathrin binding region in arrestin by promoting hydrogen bond formation between Q189 and D194. **d**, **e** Structural rearrangement located in the MEK1 interaction region (**d**) and SRC interaction (P1) region (**e**) in the arrestin2-V2Rpp-4 complex.

Elimination of the phosphate from pT360 in V2Rpp-4 not only led to the loss of phosphate binding at the V4/β4 site, but also repositioned the phospho-segment from P353 to pT360, which also eliminated phosphate binding at the V3/β3 site. Transposition of the segment encompassing the P353-to-pT360 region of V2Rpp-4 enabled the rearrangement of H-bonds between pT359 and K11 of arrestin2 and weak charge–charge interactions between pT359 and both K294 and R25 of the polar core of arrestin2, which could not be predicted by previously solved arrestin complex structures (Fig. 6a, Supplementary Fig. 7d, and Supplementary Table 12). Therefore, the binding of V2Rpp-4 represents an alternative binding mode that is not compatible with binding at either the V3 or V4 site. The site accommodating pT359 in the arrestin2-V2Rpp-4 complex structure was therefore named V3′4′. Notably, the interaction of pT359 with the V3′4′ site in the arrestin2-V2Rpp-4 complex structure was significantly weaker than the combined interactions of the V3 and V4 sites, consistent with the binding data, which showed an approximately 5-fold decrease in affinity for V2Rpp-4 compared with that for V2Rpp-FP. Moreover, the unique phospho-interaction pattern of V2Rpp-4 with arrestin also suggests that binding of the phospho-interaction sites of V3 or V3′4′ is determined by the presence of an interaction at V4, which indicates a potential interdependent phospho-interaction mechanism of receptor-arrestin interactions.

Replacement of the V3 and V4 interactions in V2Rpp-FP by V3′4′ binding of V2Rpp-4 mainly caused rotamer changes of the polar core residues L293, K294, and H295, which may correlate to conformational changes located at distant arrestin2 positions that are indicated by RMSDs (Supplementary Figs. 8g, 8h, and 9d). Residues in the arrestin2-V2Rpp-4 complex structure, including regions involved in clathrin, c-Raf-1, MEK1, and SRC binding, exhibited Cα differences of more than 1.5 Å compared with the arrestin2-V2Rpp-FP complex structure. For example, D194 formed a well-defined hydrogen bond with Q189 in the arrestin2-V2Rpp-4 complex structure, which might stabilize the overall conformation of the clathrin-binding site at this particular loop (Fig. 6b, c and Supplementary Fig. 10h). Similarly, the conformational changes located at the potential MEK1-interacting region (K355-E359) and the SRC-binding region (A90-K94, the P1 site)^4,36^ were also obvious (Fig. 6d, e and Supplementary Fig. 10i, j).

Importantly, the ^1^H NMR spectrum of K357TMSiPhe in response to V2Rpp-4 incubation was quite analogous to that of V2Rpp-6-7, but significantly different from the spectrum recorded for V2Rpp-FP or V2Rpp-1 (Fig. 4f). These NMR spectrum patterns were correlated with the cellular effects of the decreased interaction between arrestin2 and MEK1 by either the T360A (V4 site) or S363A/S364A (V6 or V7 site) mutation in V2R (Fig. 4h). Therefore, although M0 and M3 might reflect the inactive conformational state of the arrestin2 K357 site for interaction with MEK1, the M2 state might be connected to the active K357 conformation for MEK1 binding. Similarly, V2Rpp-4 did not induce the active conformational state of R307 in the ^1^H NMR spectrum, which correlated with the effect of the T360A mutation of V2R on the c-Raf-1 and arrestin2 interaction in response to vasopressin stimulation observed in cellular studies (Fig. 3e, g).

## Discussion

Deciphering the principles underlying receptor phosphorylation-coded arrestin conformations and functions lies at the heart of GPCR biology and biased signaling. Previous crystallographic, Cryo-EM and NMR studies have identified multiple potential phospho-binding pockets located on the arrestin surface, and different receptor phospho-binding patterns are correlated with selective arrestin functions and conformational states^4,6,7,18–21^. However, the contributions of each individual phospho-residue binding to a specific site to the affinity, conformational state and particular functions of arrestins have not been elucidated. In this study, we addressed this question by systematically investigating the binding affinities, crystal structures, NMR spectra and functional characterizations with downstream effectors of a series of phosphopeptides derived from the V2 receptor phospho-C-tail, which differed by only one or two phospho-residues from the fully phosphorylated V2 receptor C-tail. We employed the genetic code expansion technique to incorporate the unnatural amino acid Cou into arrestin2^31^, which enabled us to determine the contribution of each phospho-residue binding to affinity through FRET measurement. As confirmed by the solved crystal structures, elimination of the specific phosphate in V2Rpp-1 or V2Rpp-3 did eliminate the phospho-binding pocket V1 or V3/β3 interaction with arrestin2, each of which led to a decrease of approximately 3-fold in the affinity. Elimination of the two phospho-interactions at the distal binding positions in arrestin2, the V6/β6 and V7/β7 positions, caused a significant 17-fold decrease in the affinity. Moreover, switching the phospho-binding located at the V3/β3 and V4/β4 positions to the V3′4′ phospho-binding pocket caused a 5-fold decrease in the binding affinity. Therefore, we provided an efficient way to measure the affinities of arrestin2 in response to the binding of different receptor phospho-binding patterns. It’s worth to note that the binding poses of the V2Rpp-1, V2Rpp-3, and V2Rpp-67 are quite similar to the V2Rpp-FP and there are structural differences at the remote arrestin2 functional sites. Then, the binding affinity changes of the bindings of phosphopeptides of the V2Rpp-1, V2Rpp-3, and V2Rpp-67 compared to that of the V2Rpp-FP most reflected the summary of changes of the enthalpy of the phospho-binding sites and the changes of entropies of the arrestin2. Because the binding mode of the V2Rpp-4 is different from that of the V2Rpp-FP, the affinity change of the binding of the V2Rpp-4 compared to that of the V2Rpp-FP reported both the enthalpy and entropy changes of the V2Rpp and the arrestin2. Our results confirmed that phospho-binding at selective arrestin sites effectively contributed to the affinity.

Importantly, we identified a different phospho-segment binding mode in the arrestin2-V2Rpp-4 complex structure. Compared with binding with V2Rpp-FP, the elimination of the phosphate from pT360 was expected to abolish V4/β4 binding but maintain the V3/β3 interaction. However, our crystal structure revealed that elimination of the pT360 interaction at the V4/β4 site caused pS357 to flip outward, which eliminated V3/β3 binding and repositioned pT359 to enable phospho-interactions at the V3′4′ site. The overall phospho-segment ranging from P353 to pT360 was reorganized. Notably, such a binding mode could not be proposed based on simple sequence analysis or deduced from previously solved arrestin structures or computational simulations, which highlights the importance of our previous hypothesis that the specific phospho-binding of arrestin, but not the primary sequence, is the determinant of specific arrestin conformations and functions^6,9^. Moreover, this result indicates that phospho-binding at individual sites could be interdependent; that is, the binding of pT360 at the V4/β4 site is a determinant of the selective interaction at the V3/β3 or V3′4′ site. If phospho-binding to each site of arrestin could be regarded as an independent variant, the combination of the receptor phospho-tail-induced arrestin conformations or functions could be easily deduced mathematically, but the presented V2Rpp-4 binding mode indicates that this is not the case. The identification of crosstalk between different phospho-interactions in arrestin by receptor phosphobarcodes could serve as an important arrestin regulatory mechanism.

With the exception of the structure of the arestin2-V2Rpp-4 complex, the structures of other arrestin2-V2R-phosphopeptide complexes revealed quite similar binding modes compared with that of arrestin2-V2Rpp-FP, and the selective absence of corresponding phospho-interactions could be predicted from the primary V2Rpp sequence. The overall arrestin2-V2Rpp structures were hallmarked by several common characteristics, including disruption of the polar core and twisting between the C-lobe relative to the N-lobe. These results supported the notion that the phospho-binding-induced activation of arrestin exhibited common mechanisms. However, detailed structural comparisons revealed subtle structural changes at the local phospho-interaction sites and specific conformational rearrangements in several remote functional regions of arrestin2 in these complex structures. These differences highlighted that different phospho-binding patterns might cause diverse conformational states of arrestin2, which might be related to selective arrestin functions. Moreover, it is known that different GRKs phosphorylate V2R at different sites to generate distinct phosphorylation patterns^37^. In particular, Gβγ subunits are known to activate GRK2 and GRK3 in addition to their roles in constituting G protein trimers^11,13,38^. Future detailed analysis of the V2R phospho-pattern generated by different GRKs and corresponding structural studies could build the link between these GRKs and selective arrestin functions downstream of V2R.

It is also worth-noting that the crystal structures of arrestin2 presented here are complexes of arrestin2 with synthetic receptor-C-tail-phosphosegments and not with phosphoreceptors^30^. The four recently solved receptor-arrestin complex structures represent the snug conformation^14–17^, which shows quite different binding modes from the arrestin2- receptor-C-tail-phosphosegments. However, five of the seven phospho-binding pockets were identical between these phosphoreceptor-arrestin snug complexes and the V2R-phosphopeptides, and these encompass phospho-binding pockets from V3/β3 to V7/β7. Therefore, the knowledge derived from structural and functional studies of arrestin2-V2Rpp-3, arrestin2-V2Rpp-4, and arrestin2-V2Rpp-6-7 could have broad value for both the snug and hanging modes, which might further participate in distinct functions, including receptor desensitization and interactions with signal transducers such as c-Raf-1/MEK1 (Supplementary Fig. 17). In addition, these findings do not exclude the possibility that the contribution of the interaction between the phosphoreceptor C-tail and the V1 site might reflect certain structural information of the phosphodecisive mechanism of arrestin in the hanging mode. Future cryo-EM or crystal structures of receptor-arrestin structures with different phosphobarcodes in both the snug and hanging conformational states could help test these hypotheses. Among the diverse arrestin activities, desensitization is one of the most important functions of arrestin in regulating GPCR activities. Importantly, not only the affinity differences but also the distinct conformational states of arrestin induced by distinct phosphoreceptor patterns contributed to the desensitization function of arrestin. Previous structural and signaling studies have suggested that the “finger loop” of arrestin actively participates in receptor association by competing with the G protein interaction and thus plays key roles in arrestin-mediated receptor desensitization^20,39–41^. Using a finger loop-deletion mutant (FLD mutant) of arrestin2, recent studies have indicated that the receptor-phospho-C-tail is not directly involved in receptor desensitization^16^. Here, we found that the binding of different phosphobarcodes to arrestin induced selective conformational changes at receptor–interaction sites, including the finger loop region. In addition, certain phosphobarcodes (encompassing the V1 site), but not others, could directly interact with the finger loop of arrestin and thus compete with the receptor core interaction. Therefore, our results suggest that the phosphobarcodes actively participate in the desensitization of the receptor by arrestins.

Because Fab30 might stabilize the arrestin2-phosphopeptide structures at a preferred conformational state, we used our recently developed DeSipher approach to examine the different conformational states in the functional sites of arrestin that have been identified in the crystal structures^7^. The NMR spectrum of the conformational variation of these sites is not only consistent with the crystallographic observations, but also linked to specific cellular functional tests. In particular, the potential active conformational states of the c-Raf-1-binding and MEK1-binding sites could be assigned in the ^1^H NMR spectrum. For example, both V1 and V3/β3 binding contributed to the full size of the S2 peak of the R307TMSiPhe probe, whereas elimination of the V6/β6–V7/β7 or switching to the V3′4′ binding interaction significantly diminished the S2 peak, which was linked to the extent of c-Raf-1 activation downstream of arrestin. In contrast, V3 site binding, but not V1 site binding, is needed for the active conformational states of K357TMSiPhe, which is a site connected to MEK1 activation. It is worth noting that the contributions of V1 site binding and V3 site binding to the binding affinity were very similar (3-fold in both cases). Therefore, in addition to the binding strength, these results indicated that the binding positions of the phosphoresidues are the key determinants for phosphodecisive arrestin functions.

In conclusion, our results indicated that the individual phospho-binding of specific arrestin sites contributed to the affinity and could induce conformational changes at remote positions of arrestin, and that these changes are connected well with their selective functions. The individual arrestin phospho-binding pockets could be interdependent, and crosstalk between different phospho-binding pockets could be a general mechanism for signaling downstream of arrestin. The phospho-binding pattern could not be simply derived from the primary sequence, and this pattern, in addition to the strength of the phospho-arrestin interactions, is the main mechanism determining the specific conformations and selective functions of arrestin.

## Methods

### Constructs

The gene of wild-type bovine arrestin2 was subcloned into the NdeI/XhoI sites of the pET22b vector with the C-terminal His tag. The mutations of arrestin2 K49TAG, F244TAG, R307TAG, K357TAG, 1-382 truncated, the mutants of pcDNA3.1-Flag-V2R, the mutants of pcDNA3.1-c-Raf-1 and the mutants of pcDNA3.1-MEK1 were generated using the Quikchange mutagenesis kit (Stratagene). The pcDNA3.1-Flag-V2R-Rluc and its mutants were created by in-fusion the gene of Rluc with the pcDNA3.1-Flag-V2R construct. The pcDNA3.1-arrestin2-YFP was created by in-fusion the gene of the YFP with the pcDNA3.1-arrestin2 construct. All constructs and mutations were verified by DNA sequencing. All sequences were aligned by MUSCLE 3.8.31 software. All primers used in our study were shown in Supplementary Table 13.

### Expression and purification of arrestin2

The gene encoding arrestin2 was cloned into the pET22b vector that contains 6× his tag in the carboxyl terminus and then was transformed into BL21(DE3) *E. coli* cells (Thermo, catalog number: EC0114). The large-scale expression cultures in LB medium were grown to an OD = 0.8 at 37 °C. The cells were induced with 300 μM IPTG for 12 h at 25 °C. Cells were collected by centrifugation and re-suspended in buffer A (20 mM Tris-HCl, pH = 8.0, 150 mM NaCl). Cells were sonicated and the cell lysate was pelleted by centrifugation. The supernatant was collected and incubated with Ni-NTA agarose beads for 2 h at 4 °C and washed with buffer B (20 mM Tris-HCl, pH = 8.0, 150 mM NaCl, 20 mM imidazole). The arrestin2 protein was eluted with the buffer C (20 mM Tris-HCl, pH = 8.0, 150 mM NaCl, 300 mM imidazole). These proteins were subsequently purified by size exclusion column (Superdex 200 increase 10/300 GL; GE Healthcare) using the UNICORN 5.20. software and the protein was exchanged to buffer D (20 mM HEPES, pH = 7.5, 150 mM NaCl).

### Expression and purification of Fab 30

The plasmid containing the gene for the 6× his tagged heavy and light chains of Fab30 cloned in the pETDuet-1 vector and was transformed into BL21(DE3) *E. coli* cells (Thermo, catalog number: EC0114). The cultured cells were then grown to an OD600 = 0.8 at 37 °C and induced with 500 μM IPTG at 18 °C for 16 h in the LB medium cultures. These cells were harvested by centrifugation and the cell pellets were lysed in buffer A (20 mM Tris-HCl, pH = 8.0, 150 mM NaCl). The solution was poured into 50 ml centrifuge bottles and spin in SLA 1500 rotor for 30 min at 20,000×*g*. All remaining purification steps were carried out in cold room. The supernatant of the cell lysate was incubated with Ni-NTA beads by 2 h at 4 °C. The beads were packed in a column and washed with 40 CV of cold buffer B (20 mM Tris-HCl, pH = 7.55, 150 mM NaCl), and then eluted with buffer C (20 mM Tris-HCl, pH = 7.55, 150 mM NaCl, 250 mM imidazole). Dialyzed with 20 mM Tris-HCl, pH = 7.55, 100 mM NaCl (buffer D) overnight and flash frozen with 10% glycerol.

### Peptide synthesis

The phosphorylated 29 amino-acid carboxy-terminal peptides corresponding to the sequence of the C-terminal of the human V2 vasopressin receptor (V2Rpp-FP:^343^ARGRpTPPpSLGPQDEpSCpTpTApSpSpSLAKDTSS^371^; V2Rpp-1:^343^ARGRTPPpSLGPQDEpSCpTpTApSpSpSLAKDTSS^371^; V2Rpp-3:^343^ARGRpTPPpSLGPQDESCpTpTApSpSpSLAKDTSS^371^; V2Rpp-4:^343^ARGRpTPPpSLGPQDEpSCpTTApSpSpSLAKDTSS^371^; V2Rpp-6-7:^343^ARGRpTPPpSLGPQDEpSCpTpTApSSSLAKDTSS^371^) were synthesized from Tufts University Core Facility.

### Differential scanning fluorimetry (DSF)

DSF is used to measure protein stability as previously reported^32,42^. DSF assays were performed in 96-well PCR plates using an RT-PCR machine (CFX96, Bio-Rad). The arrestin2, phospho-peptide/arrestin2/ complex, and phospho-peptide/arrestin2/Fab 30 complex were incubated with BODIPY dye at room temperature for 30 min in a buffer (20 mM HEPES, 150 mM NaCl, pH = 7.5), respectively. The final concentration of each protein was 1 μM and the final probe concentration was 1 μM of BODIPY dye. All reactions were incubated at room temperature for 30 min before scanning in the PCR machine. The fluorescence was measured in 1 °C temperature intervals from 20 to 90 °C by using the SYBR Green filter set. The multicomponent data was exported on a Microsoft Excel sheet. To calculate Tm values, more accurate fitting to the Boltzmann equation can be performed using Graphpad prism software.

### FRET assay for dissociation constant determination

Steady state fluorescence was measured by a Varioskan flash (Thermo Scientific) instrument at 25 °C. Typically, 80 μl of samples containing 1 μM arrestin2 L293Cou with 1 μM Fab30 incubated with rising concentrations of different phosphopeptides in a quartz microplates 96-wells were excited at 280 nm, and the emitted fluorescence was measured from 300 or 380–550 nm using a 1 nm step size. Fluorescence intensity analysis of normalized ratio F450/F330 was calculated from relative fluorescence intensity at 450 and 330 nm. The dissociation constant was determined by fitting to the nonlinear regression equation *y* = Bmax [X] / (Kd + [X]) + NS[X] + Background as ours previously reported^31^.

### Preparation crystallization

arrestin2 (20 μM) was incubated with excess phosphopeptides for 30 min at 25 °C, then excess of Fab30 was added and the complex was incubated for 1 h at 25 °C. The arrestin2-phosphopeptides-Fab30 complex was purified by size exclusion chromatography (Superdex 200 10/300 GL; GE Healthcare) in buffer including 20 mM HEPES pH 7.5, 150 mM NaCl and 1 mM TCEP. The purified complex was concentrated to 10 mg/ml. Crystals of arrestin2-V2Rpp-3-Fab30 complex were grown in hanging drops containing 1.5 μl of complex solution and 1.5 μl of a well solution composed of 15% PEG 3350, 0.1 M HEPES, pH = 7.5 and 0.2 mM DMSO at 16 °C. Crystals of arrestin2-V2Rpp-1-Fab30 complex, arrestin2-V2Rpp-4-Fab30 complex, arrestin2-V2Rpp-6-7-Fab30 complex were grown in hanging drops containing 1.5 μl of complex solution and 1.5 μl of a well solution composed of 10% PEG 3350, 0.1 M Succinic acid, pH = 6.5–7.5 and 0.2 mM DMSO at 16 °C. The crystal appeared after about three days. Crystals of arrestin2-V2Rpp-3-Fab30 complex were flash frozen in liquid nitrogen after a 30 s soak in 17% PEG 3350, 0.1 M HEPES, pH = 7.5, 0.2 mM DMSO and 20% ethylene glycol. Crystals of arrestin2-V2Rpp-1-Fab30 complex, arrestin2-V2Rpp-4-Fab30complex, arrestin2-V2Rpp-6-7-Fab30 complex were flash frozen in liquid nitrogen after a 30 s soak in 12% PEG 3350, 0.1 M Succinic acid, pH = 7.0, 0.2 mM DMSO and 20% ethylene glycol.

### Data collection and structure determination

X-ray diffraction data of all complexes were collected at 100 K on beamline BL19U1 of the Shanghai Synchrotron Radiation Facility (SSRF, Shanghai, China) using Blu-Ice modified based on version 5.0 and a Pilatus 6M detector (Dectris). All data collected were indexed, integrated and scaled using software of XDS (version 0.89) and Aimless within CCP4 Interface (version 7.0.078) respectively^43,44^. The structures of all complexes were solved by molecular replacement using arrestin2-V2Rpp-FP-Fab30 complex (PDB code: 4JQI) as a search model by Phaser within PHENIX package (version 1.14-3260-000)^45^. Structural refinement was carried out by phenix. refine. In the refinement process, the WinCoot 0.8.9 was used for the model adjustment, and water finding. The structure models were checked using the PROCHECK. Structural figures were prepared using Pymol (version 1.7). The relevant PDB codes and hyperlink about the arrestin2-V2Rpp-1-Fab30 complex, arrestin2-V2Rpp-3-Fab30 complex, arrestin2-V2Rpp-4-Fab30 complex and arrestin2-V2Rpp-6-7-Fab30 complex were provided in “Data availability” section.

### Accessible solvent area calculation of corresponding functional regions in arrestin2

FreeSASA 2.0.3. python package was used to calculate the solvent accessible surface area for chosen corresponding functional regions in arrestin2^46^ (NLS: P45-R51; P1: S86-P91; clathrin: R188-S193 and K326-G332; Receptor: I241-A247; c-Raf-1: S302-E308; MEK1: L351-K357). To be specific, we employed the implemented Shrake–Rupley algorithm in freeSASA with the following parameters: *n*-points = 100, probe-radius = 1.4 Å.

### Expression and purification of arrestin2 TMSiPhe mutations

The pEVOL TMSiPheRS plasmids encoding specific *M. jannaschii* tyrosyl amber suppressor tRNA/tyrosyl-tRNA synthtase mutants were co-transformed into BL21 (DE3) *E. coli* cells (Thermo, catalog number: EC0114) together with the pET22b vector harboring the target arrestin2 mutant. The *E. coli* cells were cultured in LB medium. After the 1 l cell culture reached OD_600_ = 0.6–0.8, the cells were induced with 300 μM IPTG and 0.2% l-arabinose for 12 h at 25 °C to allow protein expression in presence of 0.5 mM TMSiPhe in the culture medium. The cells were lysed by French pressing in buffer A (50 mM Tris-HCl, pH = 8.0, 150 mM NaCl) and the lysate was batch binding with 300 μl Ni-NTA column (GE Healthcare, USA). After an extensive washing with buffer A, the target protein was eluted using 300 mM imidazole in buffer A. The arrestin2 contains 6× his tag in the carboxyl terminus, thus enabled discarding the incomplete protein during the purification of this step. These proteins were subsequently purified by size exclusion column (Superdex 200 increase 10/300 GL; GE Healthcare) and the buffer was exchanged to buffer B (20 mM HEPES, pH = 7.5, 150 mM NaCl), and the purified protein was concentrated to 5 mg/ml. Finally, the protein was flash frozen in liquid nitrogen and stored at −80 °C.

### GST pull down assay

In total, 0.1 μM wild-type or mutant arrestin2 was mixed with 0.5 μM phospho-receptor C-tail fragment (V2Rpp-FP) and incubated in binding buffer (20 mM Tris-HCl, pH = 7.5, 150 mM NaCl) at 25 °C for 30 min In all, 1 μM GST-clathrin was then added and incubated for 1 h. Subsequently, 10 μl GST beads were added into the mixture and the mixture was rolled at 4 °C for 2 h. The GST beads were collected by centrifuge at 1,000 × g and washed with wash buffer (binding buffer with 0.5% Tween20) for four times. After removing the supernatant, the samples were re-suspended in 50 μl 2× SDS loading buffer and boiled for 10 min before western blot. The primary antibodies including anti-GST (Cell Signaling Technology, Catalog #2622) and anti-His (Cell Signaling Technology, Catalog #2366) were used in 1:1000 dilution. The secondary antibodies including anti-rabbit (Sigma Aldrich, Catalog #A6154) and anti-mouse (Sigma Aldrich, Catalog #A4416) were used in 1:5000 dilution. The data analysis using the Image J (version 1.8.0) and Graphpad Prism 8.0 software. Uncropped and unprocessed scans of the blots were provided in Supplementary Fig. 18 and Source Data file.

### NMR experiment

Arrestin2 TMSiPhe mutants prepared for 1D ^1^H NMR spectra analysis were performed as reported previously^7^. To detect distinct phosphopeptides induced conformational changes of arrestin2, 10 μM arrestin2 TMSiPhe mutations proteins were mixed with or without 100μM phosphopeptides (V2Rpp-FP, V2Rpp-1, V2Rpp-3, V2Rpp-4, or V2Rpp-6-7) and incubated in binding buffer (20 mM HEPES, pH = 7.5, 150 mM NaCl) for 30 min at 25 °C. 1D ^1^H NMR spectra of the arrestin2 TMSiPhe mutations (10 μM) with or without the phosphopeptides (100 μM) were recorded in buffer (20 mM HEPES, pH = 7.5, 150 mM NaCl, 10% D_2_O) using a Bruker 950 MHz NMR spectrometer at 25 °C by Bruker TopSpin 3.6 software. Signal-to-noise ratio was measured using “sinocal” routine within topspin 4.0 (Bruker Biospin, Billerica MA). The data analysis using the MestReNova-6.1.1-6384 software.

### ELISA assay

To evaluate the expression level of wild-type V2R and its mutants, HEK293 cells were transiently transfected with wild-type and mutants of V2R or vehicle (pcDNA3.1) using PEI regent at in 6-well plates. After incubation at 37 °C for 18 h, transfected cells were plated into 24-well plates at a density of 10^5^ cells per well and further incubated at 37 °C in a 5% CO_2_ atmosphere for 18 h. Cells were then fixed in 4% (w/v) paraformaldehyde and blocked with 5% (w/v) BSA at room temperature. Each well was incubated with 200 μl of monoclonal anti-FLAG (F1804, Sigma-Aldrich) primary antibody overnight at 4 °C and followed by incubation of a secondary anti-rabbit antibody (A6154, Sigma-Aldrich) conjugated to horseradish peroxide for 1 h at room temperature. The anti-FLAG antibody (primary antibody) was used in 1:1000 dilution. The anti-rabbit antibody (secondary antibody) was used in 1:5000 dilution. After washing, 200 μl of TMB (3,3′,5,5′-tetramethylbenzidine) solution were added. Reactions were quenched by adding an equal volume of 0.25 M HCl solution and the optical density at 450 nm was measured using the TECAN (Infinite M200 Pro NanoQuant) luminescence counter. For determination of the constitutive activities of different V2R constructs or mutants, varying concentrations of desired plasmids were transiently transfected into HEK293 cells, and the absorbance at 450 nm was measured. Source data are provided in a Source Data file.

### BRET assay

For arrestin2-c-Raf-1 interaction by V2R regulated experiment, HEK293 cells (obtained from Cell Resource Center of Shanghai Institute for Biological Sciences (Chinese Academy of Sciences, Shanghai, China)) seeded in six-well plates were co-transfected with wild type Flag-V2R or Flag-V2R with corresponding site mutation, Rluc-arrestin2 and c-Raf-1 R125-CCPGCC-L126 that in pcDNA3.1 vector using polyethylenimine (PEI). After 24 h of transfection, the cells were detached and distributed into 96-well plates at a density of ~25,000 cells per well. After another 24 h incubation at 37 °C, the cells were labeled FlAsH-EDT2 for 40 min at 37 °C and washed twice with Tyrode’s buffer (140 mM NaCl, 2.7 mM KCl, 1 mM CaCl_2_, 12 mM NaHCO_3_, 5.6 mM d-glucose, 0.5 mM MgCl_2_, 0.37 mM NaH_2_PO_4_, and 25 mM HEPES, pH = 7.4) and stimulated with or without Argipressin (final concentration of 10 μM) at 37 °C for 10 min Luciferase substrate coelenterazine-h was added at a final concentration of 5 μM before light emissions were recorded using a Mithras LB940 microplate reader (Berthold Technologies) equipped with BRET filter sets. The BRET signal was determined by calculating the ratio of the light intensity emitted by YFP (530/20 nM) over the light intensity emitted by Rluc (485/20 nM). Source data are provided in a Source Data file.

For arrestin2-MEK1 interaction by V2R regulated experiment, HEK293 cells (obtained from Cell Resource Center of Shanghai Institute for Biological Sciences (Chinese Academy of Sciences, Shanghai, China)) seeded in six-well plates were co-transfected with wild type Flag-V2R or Flag-V2R with corresponding site mutation, Rluc-arrestin2 and MEK1 T238-CCPGCC-H239 that in pcDNA3.1 vector using PEI. 24 h after transfection, the cells were detached and distributed into 96-well plates at a density of ~25,000 cells per well. After another 24 h of incubation at 37 °C, the cells were labeled FlAsH-EDT2 for 40 min at 37 °C and washed twice with Tyrode’s buffer (140 mM NaCl, 2.7 mM KCl, 1 mM CaCl_2_, 12 mM NaHCO_3_, 5.6 mM D-glucose, 0.5 mM MgCl_2_, 0.37 mM NaH_2_PO_4_, and 25 mM HEPES, pH = 7.4) and stimulated with or without Argipressin (final concentration of 10 μM) at 37 °C for 10 min Luciferase substrate coelenterazine-h was added at a final concentration of 5 μM before light emissions were recorded using a Mithras LB940 microplate reader (Berthold Technologies) equipped with BRET filter sets. The BRET signal was determined by calculating the ratio of the light intensity emitted by YFP (530/20 nM) over the light intensity emitted by Rluc (485/20 nM). Source data are provided in a Source Data file.

For arrestin2 recruitment experiment, HEK293 cells seeded in six-well plates were transfected with 2 μg BRET donor the wild type Flag-V2R-Rluc or Flag-V2R-Rluc with corresponding phosphosite mutation and 1 μg BRET acceptor arrestin2-YFP. After 24 h of transfection, the cells were detached and distributed into 96-well plates at a density of ~25,000 cells per well. After another 24 h incubation at 37 °C, the cells were washed twice with Tyrode’s buffer (140 mM NaCl, 2.7 mM KCl, 1 mM CaCl_2_, 12 mM NaHCO_3_, 5.6 mM D-glucose, 0.5 mM MgCl_2_, 0.37 mM NaH_2_PO_4_, and 25 mM HEPES, pH = 7.4) and incubated with vehicle or different concentration ligands (Argipressin) at 37 °C for 10 min Luciferase substrate coelenterazine-h was added at a final concentration of 5 μM before light emissions were recorded using a Mithras LB940 microplate reader (Berthold Technologies) equipped with BRET filter sets. The BRET signal was determined by calculating the ratio of the light intensity emitted by YFP (530/20 nm) over the light intensity emitted by Rluc (485/20 nm). Source data are provided in a Source Data file.

### Co-immunoprecipitation (Co-IP) assay

The functions of FlAsH-tagged effectors were examined by Co-IP assay. Both FlAsH-tagged MEK1 and c-Raf-1 sensors were able to associate with the arrestin-YFP, similar to their wild type counterparts.

HEK293 cells were co-transfected with plasmids encoded the arrestin2-YFP and HA-c-Raf-1-WT or HA-c-Raf-1-R125-CCPGCC-L126; arrestin2-YFP and HA-MEK1-WT or HA-MEK1-T238-CCPGCC-H239. Forty hours after transfection, the cells were starved for 8 h and stimulated with 10 μM isoproterenol (ISO) for 15 min Cells were collected in cold lysis buffer and then centrifuged for 30 min at 12,000 × *g* after 1 h of end-to-end rotation at 4 °C. HA-MEK1/HA-c-Raf-1 was immunoprecipitated by HA-antibody-conjugated agarose, and the arrestin2 were detected by western blotting using specific YFP antibodies. The primary antibodies including anti-HA (Cell Signaling Technology, Catalog #5017) and anti-YFP (Biovision, Catalog #3991) were used in 1:1000 dilution. The secondary antibodies were used in 1:5000 dilution. Uncropped and unprocessed scans of the blots were provided in Supplementary Fig. 19 and Source Data file.

### Statistical analysis

For all experiment, the number of replicates and *P* value cutoff are described in the respective Figure legends. Error bars are shown for all data points with replicates as a measure of variation with the group. Statistical differences were determined by one-way analysis of variance using the analysis software GraphPad Prism (*P < 0.05; **P < 0.01; ***P < 0.001; #*P* < 0.05; ##*P* < 0.01; ###*P* < 0.001; *&P* < 0.05; *&&P* < 0.01; *&&&P* < 0.001; ns: no significant difference).

### Reporting summary

Further information on research design is available in the Nature Research Reporting Summary linked to this article.

## Supplementary information

Supplementary Information

Reporting Summary

## Data Availability

Data supporting the findings of this manuscript are available from the corresponding authors upon reasonable request. A reporting summary for this Article is available as a Supplementary Information file. The arrestin2-V2Rpp-1-Fab30 complex, arrestin2-V2Rpp-3-Fab30 complex, arrestin2-V2Rpp-4-Fab30 complex and arrestin2-V2Rpp-6-7-Fab30 complex crystal structures and associated diffraction data have been deposited in the Protein Data Bank with the accession codes PDB: 7DF9, PDB: 7DFC, PDB: 7DFA and PDB: 7DFB respectively. The hyperlink about the arrestin2-V2Rpp-1-Fab30 complex, arrestin2-V2Rpp-3-Fab30 complex, arrestin2-V2Rpp-4-Fab30 complex and arrestin2-V2Rpp-6-7-Fab30 complex were as follows: https://www.rcsb.org/structure/unreleased/7DF9; https://www.rcsb.org/structure/unreleased/7DFC; https://www.rcsb.org/structure/unreleased/7DFA; https://www.rcsb.org/structure/unreleased/7DFB. Source data are provided with this paper.

## Source data

Source Data
