# Supplementary material for: Structural studies of phosphorylation-dependent interactions between the V2R receptor and arrestin-2
Source: Nat Commun. 2021 Apr 22;12:2396. doi: 10.1038/s41467-021-22731-x (PMC8062632; doi:10.1038/s41467-021-22731-x)
Supplement: Supplementary file 1 — Supplementary Information [file 41467_2021_22731_MOESM1_ESM.pdf]

Supplementary information

Structural studies of phosphorylation-dependent interactions between the V2R receptor and arrestin-2

Qing-Tao He[1,2,9], Peng Xiao[1,9], Shen-Ming Huang[2,9], Ying-Li Jia[2,9], Zhong-Liang Zhu[3,9], Jing-Yu Lin[2,4,9], Fan Yang[1,2], Xiao-Na Tao[1], Ru-Jia Zhao[1], Feng-Yuan Gao[1], Xiao-Gang Niu[5], Kun-Hong Xiao[6], Jiangyun Wang[7,8*], Changwen Jin[5*], Jin-Peng Sun[1,2*], Xiao Yu[4*]

Affiliations:

[1]Key Laboratory Experimental Teratology of the Ministry of Education and Department of Biochemistry and Molecular Biology, School of Basic Medical Sciences, Cheeloo college of Medicine, Shandong University, 44 Wenhua Xi Road, Jinan, Shandong, 250012, China.

[2]Key Laboratory of Molecular Cardiovascular Science, Ministry of Education, Peking University, 38 Xueyuan Road, Haidian district Beijing, 100191, China.

[3]School of Life Sciences, University of Science and Technology of China, 96 Jinzhai Road, Hefei, Anhui 230026, China.

[4]Key Laboratory Experimental Teratology of the Ministry of Education and Department of Physiology, School of Basic Medical Sciences, Cheeloo college of Medicine, Shandong University, 44 Wenhua Xi Road, Jinan, Shandong, 250012, China.

[5]Beijing Nuclear Magnetic Resonance Center, College of Chemistry and Molecular Engineering, School of Life Sciences, Peking University, Beijing 100084, China

[6]Department of Pharmacology and Chemical Biology, School of Medicine, University of Pittsburgh, Pittsburgh, PA 15261, USA

[7]Institute of Biophysics, Chinese Academy of Sciences, 15 Datun Road, Chaoyang district, Beijing 100101, China.

[8]Shenzhen Institute of Transfusion Medicine, Shenzhen Blood Center, Futian District, Shenzhen 518052, China.

[9] These authors contributed equally: Qing-Tao He, Peng Xiao, Shen-Ming Huang, Ying-Li Jia, Zhong-Liang Zhu, Jing-Yu Lin.

* Corresponding author: Jin-Peng Sun (corresponding author)
E-mail: sunjinpeng@sdu.edu.cn
Xiao Yu (corresponding author)
E-mail: yuxiao@sdu.edu.cn
Changwen Jin (corresponding author)
E-mail: changwen@pku.edu.cn
Jiangyun Wang (corresponding author)
Email: jwang@ibp.ac.cn

Supplementary Fig. 1

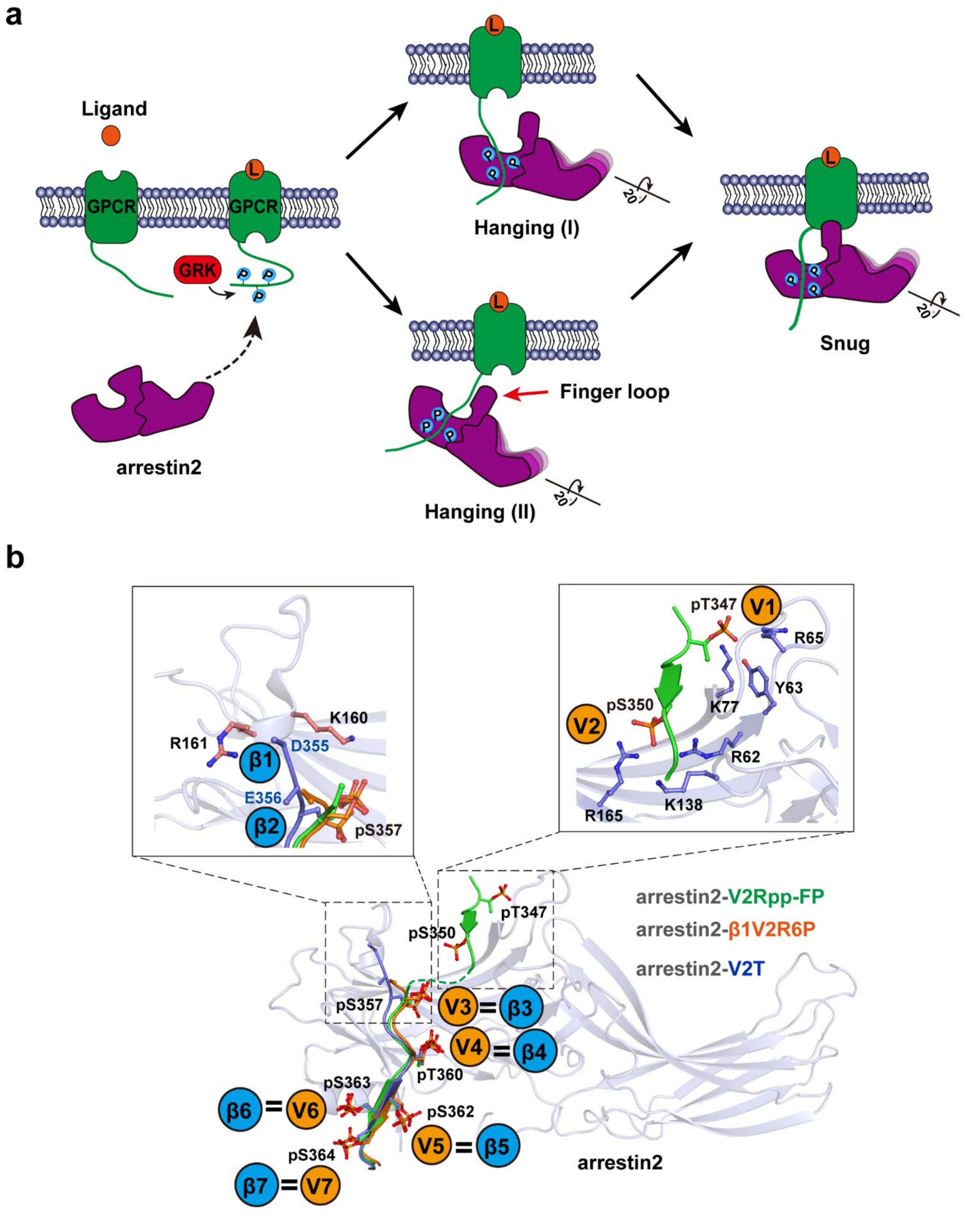

Supplementary Fig. 1. The potential phosphate binding models in arrestin2.

a). Schematic representation of two interaction modes of GPCR-arrestin2 complexes. GPCRs were phosphorylated by GRKs, which enabled the recruitment of arrestins through the phosphorylated carboxyl termini of receptors. The interaction of the receptor was solely mediated by the receptor phosphorylated C-tail, whereas the interaction of the receptor was mediated by both the phosphorylated carboxyl terminus and seven transmembrane cores, which were called the 'snug' state. In the hanging state, the receptor phosphorylated C-tail may interact with arrestin by crossing with the finger loop (lower route) or not crossing with the finger loop (upper route), as suggested by crystallographic or Cryo-EM studies[1-3]. The blue circles: phosphorylation; The orange circles: Ligand.

b). Two models of phosphate binding pockets in arrestin2. "β1-β7" represent the potential phosphate binding sites revealed by the Cryo-EM structure of the arrestin2-V2T complex (PDB:6NI2); "β2-β7" represent the potential phosphate binding sites revealed by the Cryo-EM structure of the arrestin2-β1V2R6P complex (PDB: 6TKO); and "V1-V7" were defined by the previously solved complex structure of arrestin2-V2Rpp-FP (PDB: 4JQI). Five sites of the pockets (β3-β7) overlapped with the phospho-binding pockets "V3-V7", indicating that these sites are shared by different receptor-arrestin interaction modes.

Supplementary Fig. 2

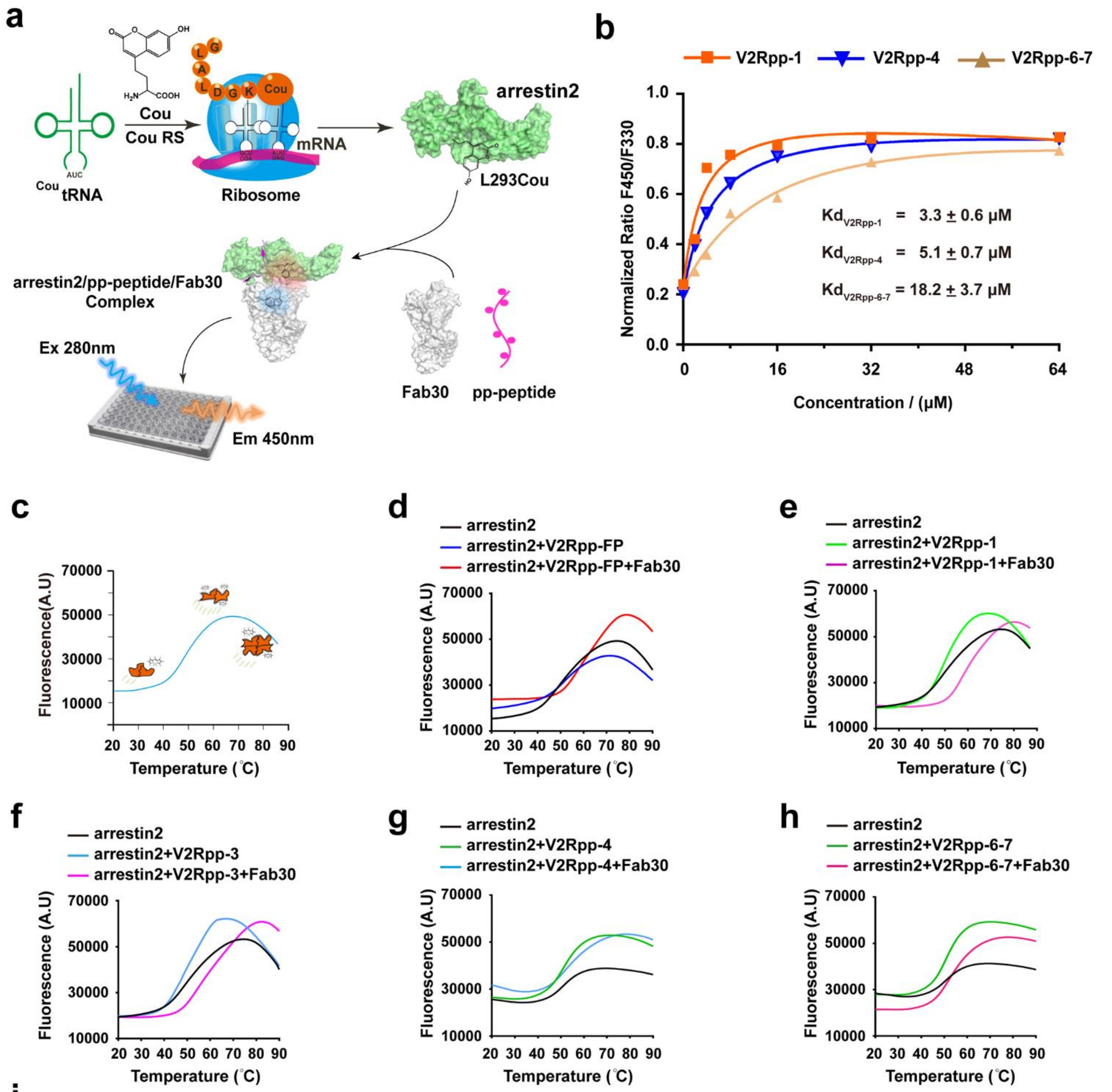

i

	V2Rpp-FP	V2Rpp-1	V2Rpp-3	V2Rpp-4	V2Rpp-6-7
arrestin2	50.06	49.16	48.70	50.92	50.68
arrestin2+Fab30	61.13	60.97	60.90	55.13	53.87
△Tm (°C)	11.07	11.81	12.20	4.21	3.19

Supplementary Fig. 2. Binding affinities and effects of Fab30 on the thermostability of arrestin2 in response to different phospho-peptide binding.

a). Schematic flowchart of FRET assay for dissociation constant determination of arrestin-phospho-peptide interaction.

The synthetic fluorescent amino acid L-(7-hydroxycoumarin-4-yl) ethylglycine (Cou) was genetically incorporated into arrestin2 and served as a fluorescent acceptor for the emission light at 330 nm, and tryptophan served as the donor, which was excited at 280 nm. Cou produced its characteristic emission light at 450 nm.

b). Analysis of the titration experiments monitored by FRET assay. The dissociation constant was determined by fitting to the nonlinear regression equation y = Bmax[X]/(Kd + [X]) + NS[X]+Background as previously described[4].

c). Schematic of thermal stability shift analysis of arrestin2-V2Rpp complexes. Typical fluorescence intensity versus temperature for protein (arrestin2, arrestin2-Phospho-peptide or arrestin2-Phospho-peptide-fab30 complex) unfolding in the presence of BODIPY. In the presence of a basic protein (at the baseline of the curve), a basic fluorescence intensity was excited under 504 nm light (depicted schematically by green curved arrows). As the temperature rose, the protein unfolded and exposed hydrophobic moieties or Cys residues (in grey), which were labelled covalently by the fluorescent dye. The fluorescent light of 511 nm (depicted by orange curved arrows) emitted by the dye molecules was recorded. Then, a gradual fluorescence decrease could be observed, which mainly indicated the removal of the protein due to precipitation or aggregation.

d-h). Thermal unfolding of protein complexes measured by BODIPY labelling. The original curve was fitted to the Boltzmann equation to derive the melting temperature. Measurements of arrestin2, arrestin2-Phospho-peptide, and the arrestin2-Phospho-peptide-Fab30 complex are presented in different curves.

i). The melting temperatures relevant to Supplementary Fig. 2d-2h. Thermal stability shift analysis of the arrestin2-Phospho-peptide-fab30 complex indicated a higher thermostability of this complex than the arrestin2-Phospho-peptide complex. ΔTm =11.07 °C (V2Rpp-FP), 11.81°C (V2Rpp-1), 12.20 °C (V2Rpp-3), 4.21 °C (V2Rpp-4), and 3.19 °C (V2Rpp-6-7).

Supplementary Fig. 3

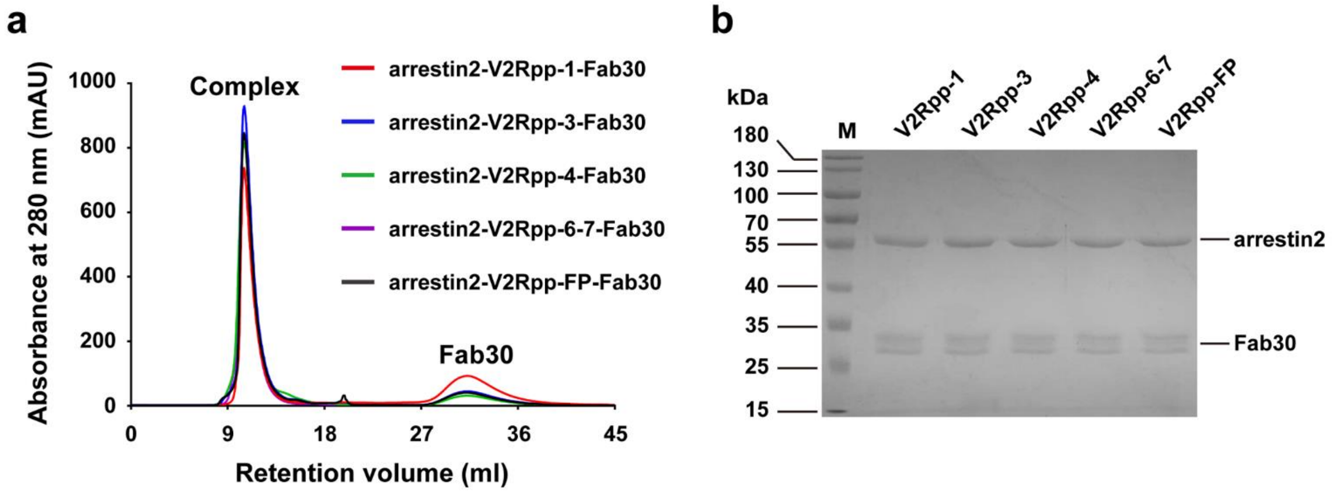

Supplementary Fig. 3. SEC analysis of arrestin2-phosphopeptide -Fab30 complex.

a). Representative elution profile of different phospho-peptide-arrestin2-Fab30 complexes by size exclusion chromatography (Superdex 200 10/300 GL; GE Healthcare).

b). Coomassie-stained gel of the size-exclusion of different phospho-peptide-arrestin2-Fab30 complexes chromatography peaks. This experiment was repeated three times (n=3) independently with similar results.

Supplementary Fig. 4

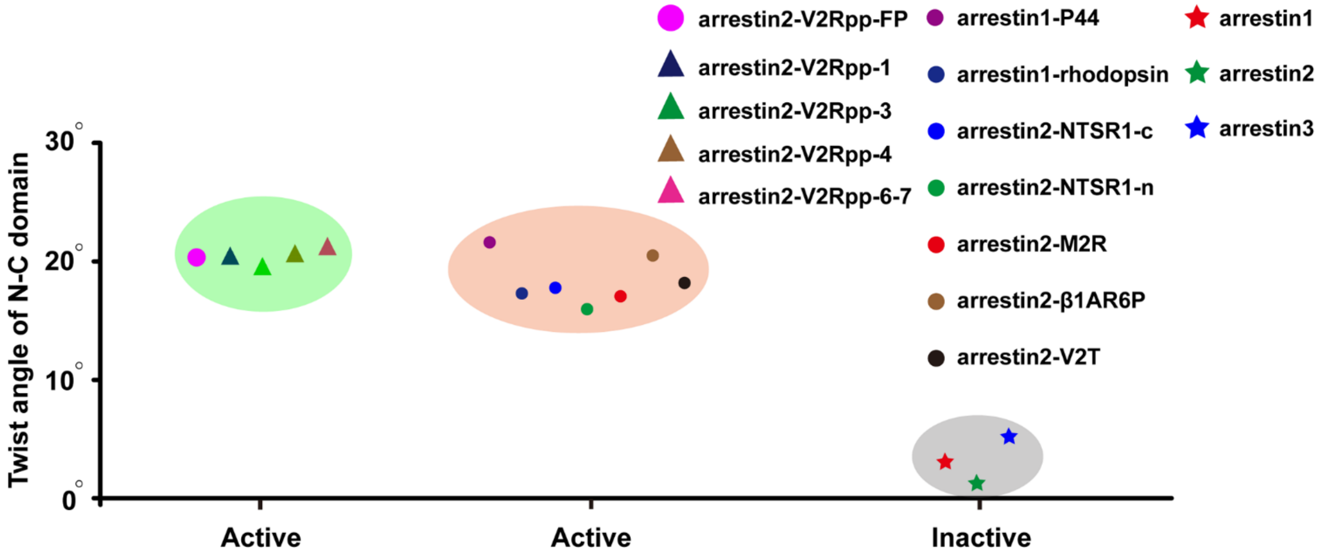

Supplementary Fig. 4. The interdomain rotation angles of the N- and C-domains in arrestin.

The interdomain rotation angle of arrestins bound to peptides or GPCRs ranged from 16° to 22° compared with inactive arrestin2. arrestin2-V2Rpp-FP, PDB: 4JQI; arrestin1-P44, PDB: 4J2Q; arrestin1-rhodopsin, PDB: 5W0P; arrestin2-NTSR1-c, PDB: 6PWC; arrestin2-NTSR1-n, PDB: 6UP7; arrestin2-M2R, PDB: 6U1N; arrestin2-β1AR6P, PDB: 6TKO; arrestin2-V2T, PDB:6NI2; arrestin1, PDB:1CF1; arrestin2, PDB:1G4M; arrestin3, PDB:3P2D.

Supplementary Fig. 5

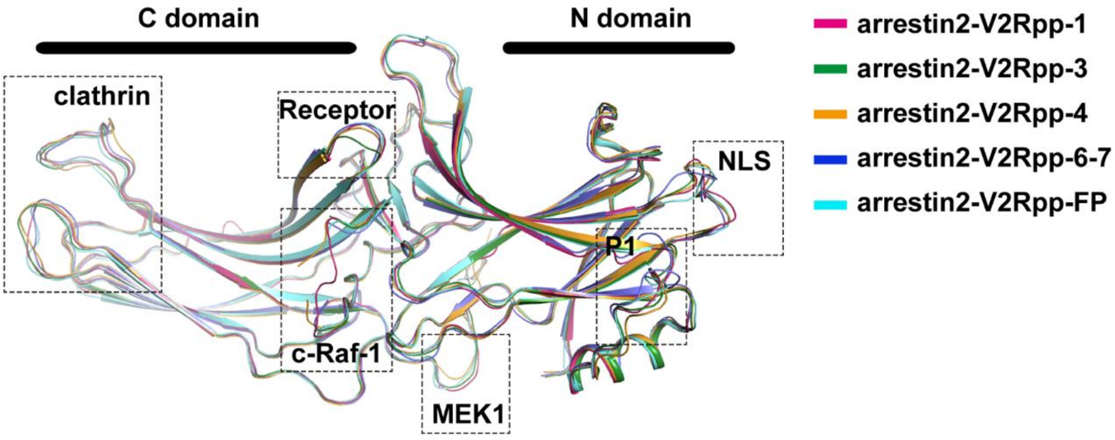

Supplementary Fig. 5. Spatial distribution of functional regions in arrestin2 matched with the solvent

exposure analysis in Figure 2e.

Supplementary Fig. 6

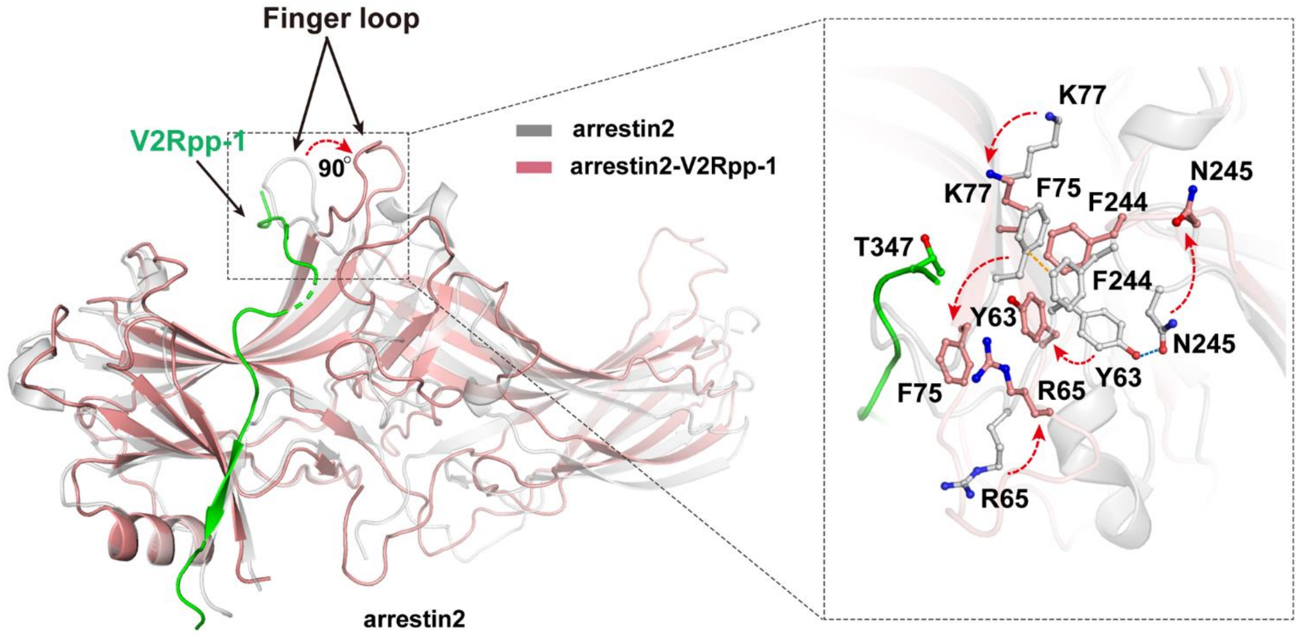

Supplementary Fig. 6. Structural rearrangement of the finger loop in the arrestin2-V2Rpp-1 complex.
Left panel: the finger loop appeared at nearly 90° with respect to inactive arrestin2 (PDB:1G4M, grey). Right panel: residue K77 turned approximately 120° to T347 of V2Rpp-1. The hydrophobic interaction of F75 and F244 was broken, and F75 rotated approximately 150° to form cation-π interactions with R65 in the arrestin2-V2Rpp-1 complex. Meanwhile, both Y63 and R65 reorganized to orient towards V2Rpp-1 but did not form strong interactions with T347 and the polar interaction between Y63 and N245 of C loop was broken.

Supplementary Fig. 7

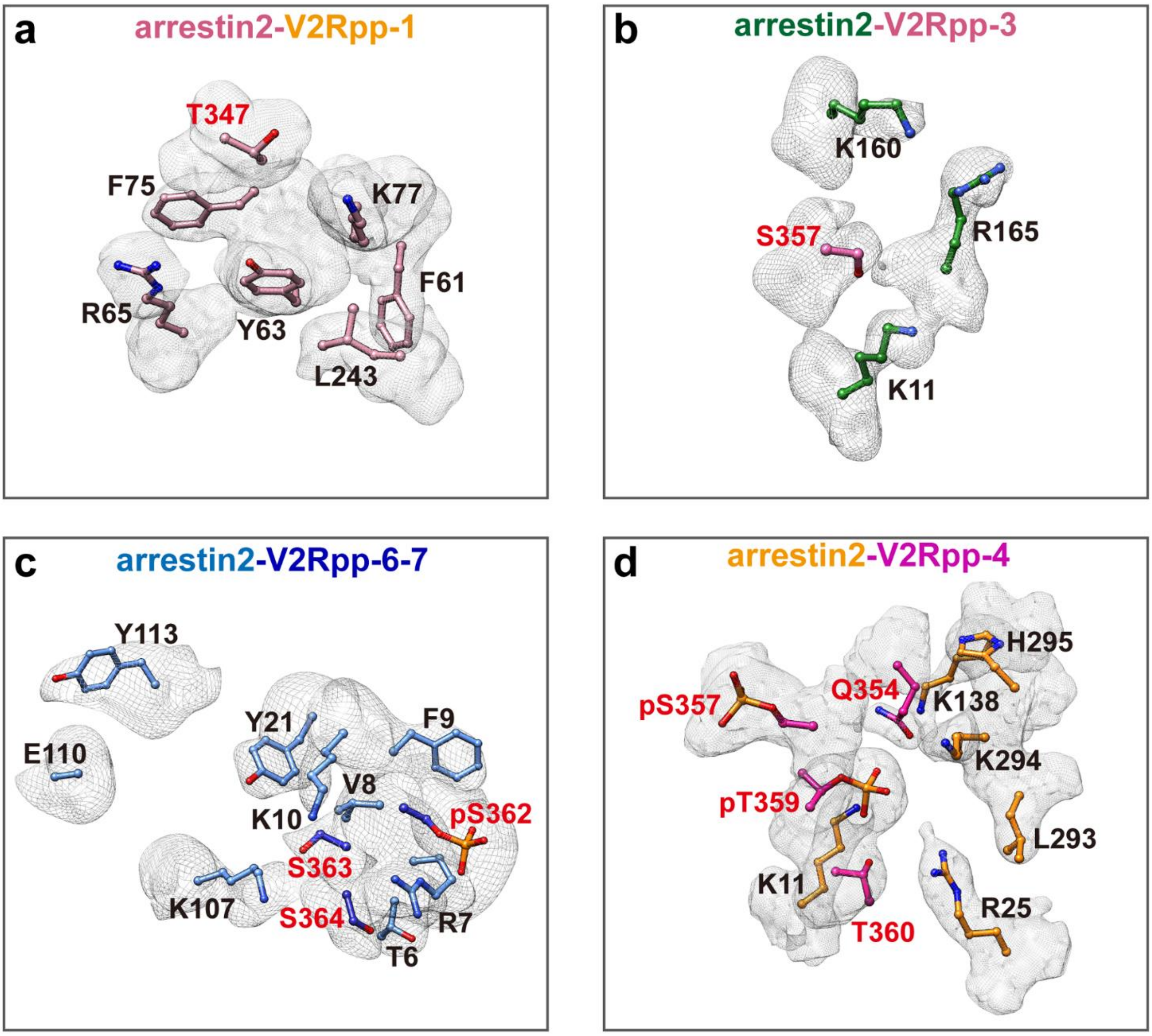

Supplementary Fig. 7. The 2Fo-Fc map of the interaction regions surrounding the phospho-deficient site of each specific V2R-phospho-peptide compared to that of V2Rpp-FP.

a-d clearly showed the electron density that are correspond to the figure 3a, figure 4a, figure 5a, figure 6a respectively.

Supplementary Fig. 8

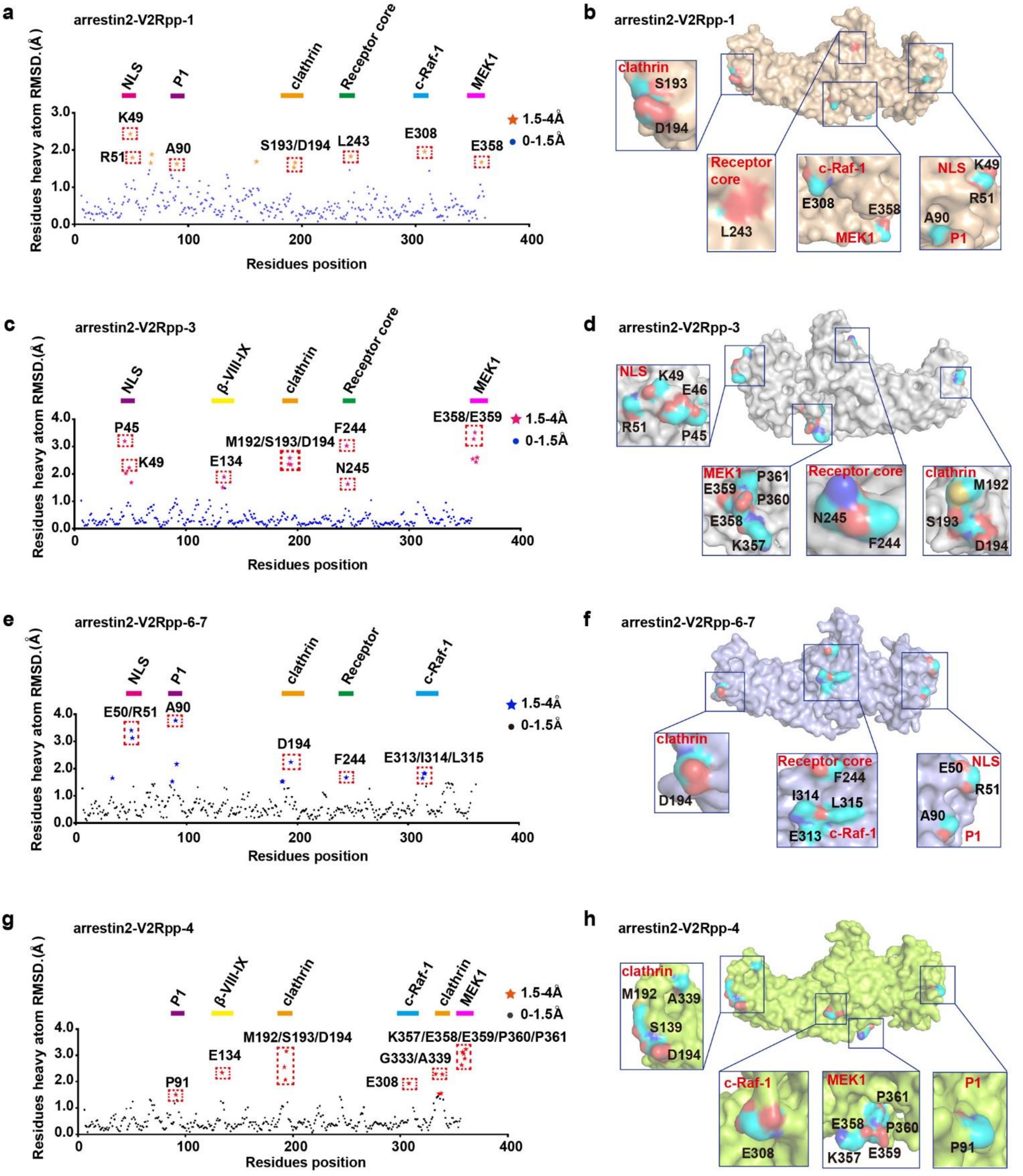

Supplementary Fig. 8. Conformational differences between different arrestin2 structures.

Plots of the distance root mean square deviations (RMSDs) for individual residues between the newly solved arrestin2-phospho-peptide complexes and previously reported arrestin2-V2Rpp-FP complexes were shown.

Important residues with significant conformational change are highlighted.

a,c,e,g): The vertical axis shows all heavy-atom RMSDs per arrestin residue, whereas the horizontal axis represents the position of each residue in arrestin2. Residue Cα deviations of 1.5 Å and above are marked with stars, and the related functional regions are annotated over the corresponding residues. 8 residues of NLS, clathrin, c-Raf-1 and MEK1 functional regions in the arrestin2-V2Rpp-1 complex (a-b), 14 residues of NLS, clathrin, receptor and MEK1 binding regions in the arrestin2-V2Rpp-3 complex (c-d), 12 residues of NLS, P1, c-Raf-1 and MEK1 functional regions in the arrestin2-V2Rpp-6-7 complex (e-f) and 14 residues related to clathrin, c-Raf-1, MEK1 and SRC binding in the arrestin2-V2Rpp-4 complex (g-h) have a main chain Cα coordinate change between 1.5 Å and 4 Å. b, d, f, h): residues highlighted in a, c, e, g were also highlighted in the surface representation.

Supplementary Fig. 9

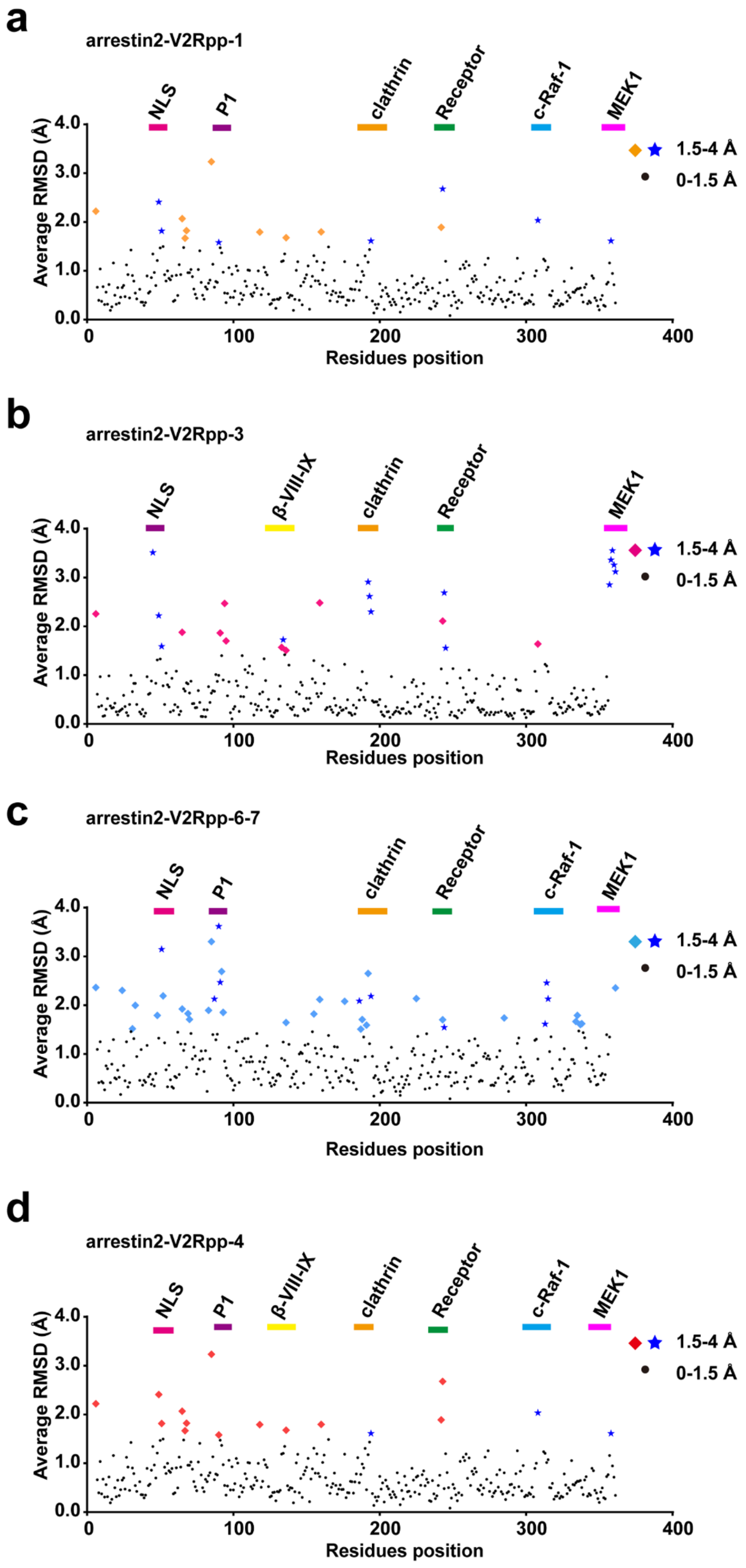

Supplementary Fig. 9. Examination of the average plots of the distance root mean square deviations (RMSDs) for residues between four newly solved arrestin2 complexes and previously reported arrestin2-V2Rpp-FP complexes.

a-d). The vertical axis shows average RMSDs per arrestin residue including all atoms, whereas the horizontal axis represents the position of each residue in arrestin2. Residue average RMSD deviations of 1.5 Å and above are marked with diamond or stars, in which stars represent residues overlapping to those found by heavy-atom RMSD analysis (Supplementary Fig. 8) whereas diamonds indicated residues that are not identified by heavy-atom RMSD analysis. The related functional regions are annotated.

Supplementary Fig. 10

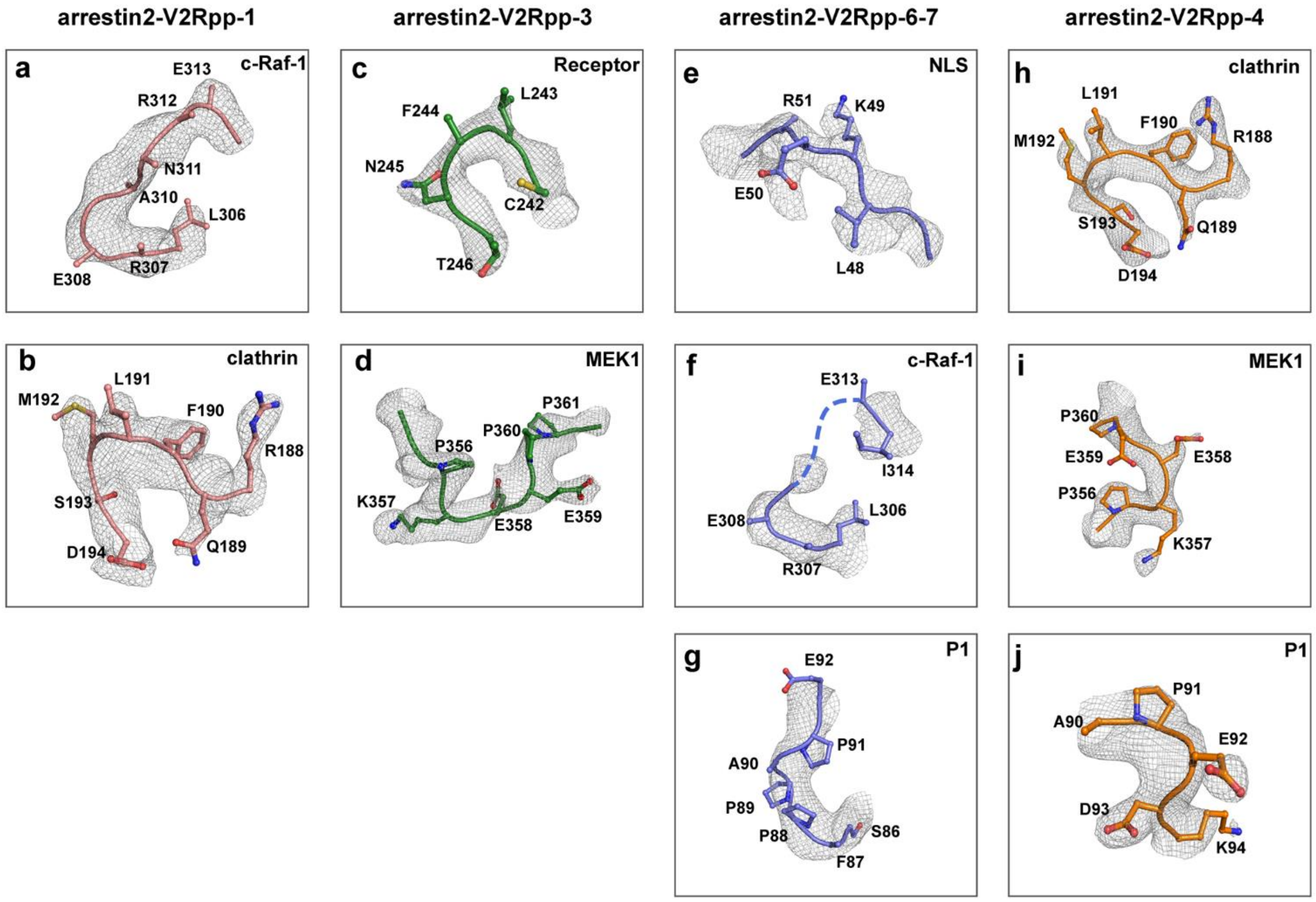

Supplementary Fig. 10. The map of the functional related regions in arrestin2.

The 2Fo-Fc annealing omits maps of arrestin2-V2Rpp-1, arrestin2-V2Rpp-3, arrestin2-V2Rpp-4 and arrestin2-V2Rpp-6-7 clearly show the electron density of the functional related region in arrestin2 that were mentioned in the main text. All maps were contoured at 1.0 σ.

Supplementary Fig. 11

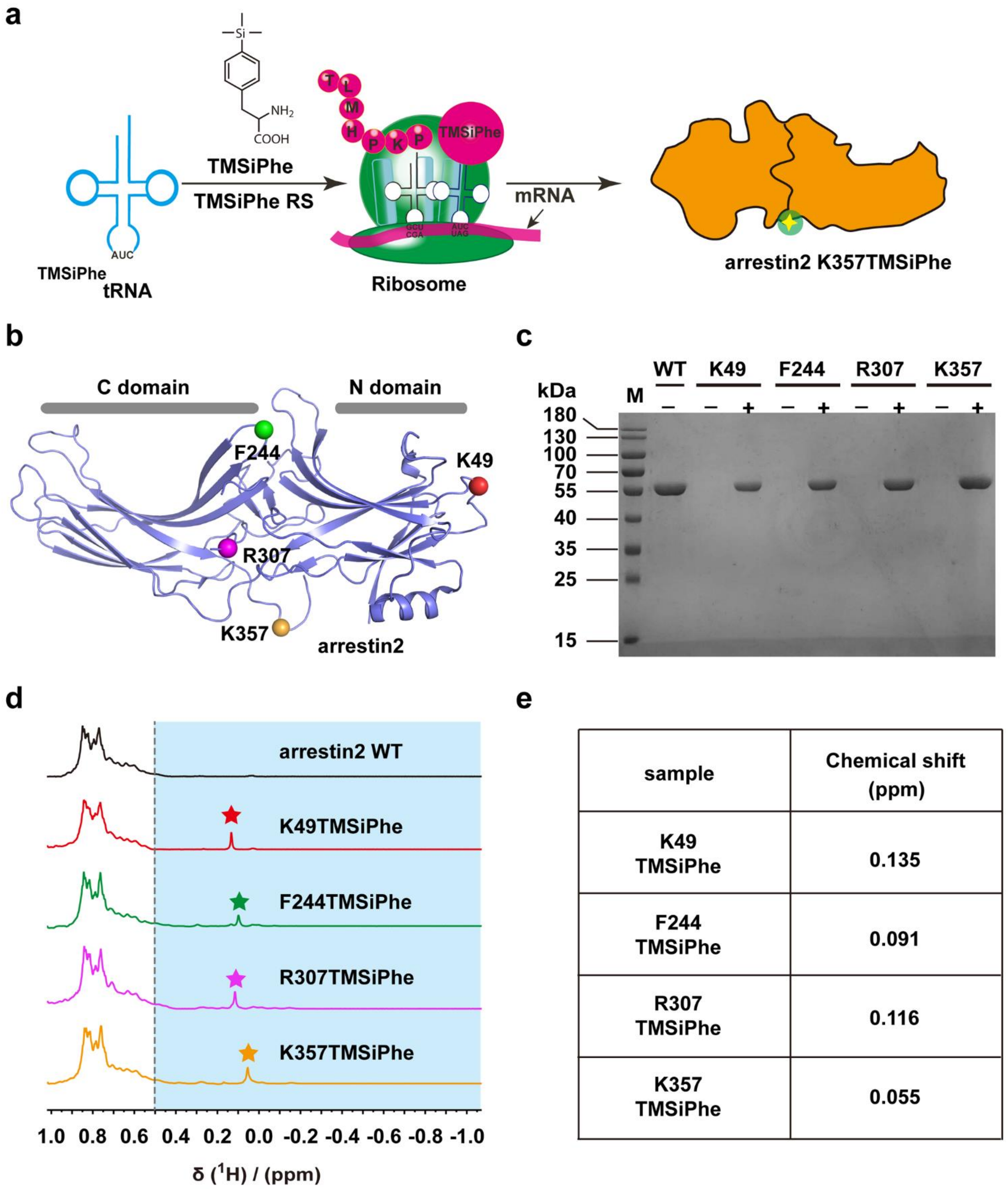

Supplementary Fig. 11. Incorporation of TMSiPhe at functionally relevant motifs of arrestin2.

a). Schematic representation of the strategy for incorporating TMSiPhe into arrestin2 as previously described. The codons that encode K49, F244, R307 and K357 were mutated to TAG, and then, *E. coli* cells cotransformed with plasmids encoding the arrestin2 mutants and the specific *M. jannaschii* tyrosyl amber suppressor tRNA/tyrosyl-

tRNA synthetase mutants (TMSiPheRS) were cultured in Luria−Bertani (LB) medium containing 0.5 mM TMSiPhe, and the expressed arrestin2 was purified by Ni-NTA affinity chromatography.

b). Frontal view of the TMSiPhe incorporation sites depicted by spheres in the active arrestin2 crystal structure (PDB: 4JQI). The red ball: K49 in the NLS region; The green ball: F244 in the C loop; The purple ball: R307 in the c-Raf-1 binding region; The orange ball: K357 in the MKE1 interaction area.

c). Coomassie-stained gel was used to analyze the purified wild-type arrestin2 or arrestin2 mutants with TMSiPhe incorporation. This experiment was repeated three times (n=3) independently with similar results. +: Presence of 1 mM TMSiPhe in the culture medium; -: Absence of 1 mM TMSiPhe in the culture medium.

d). 1D ^{1}H NMR spectra of arrestin2 labelled described in (Supplementary Fig. 11b). The spectra were recorded with 10 μM TMSiPhe-incorporated arrestin2, and the total recording time per spectrum was 15 min. The chemical shift for analysis was less than 0.55 ppm. The pentagrams: the position of NMR signal peak for the arrestin2 inserted TMSiPhe at different sites. Red: K49; green: F244; pink: R307; orange: K357.

e). Chemical shifts in the 1D ^{1}H-NMR spectra assigned to different positions incorporated with TMSiPhe in arrestin2.

Supplementary Fig. 12

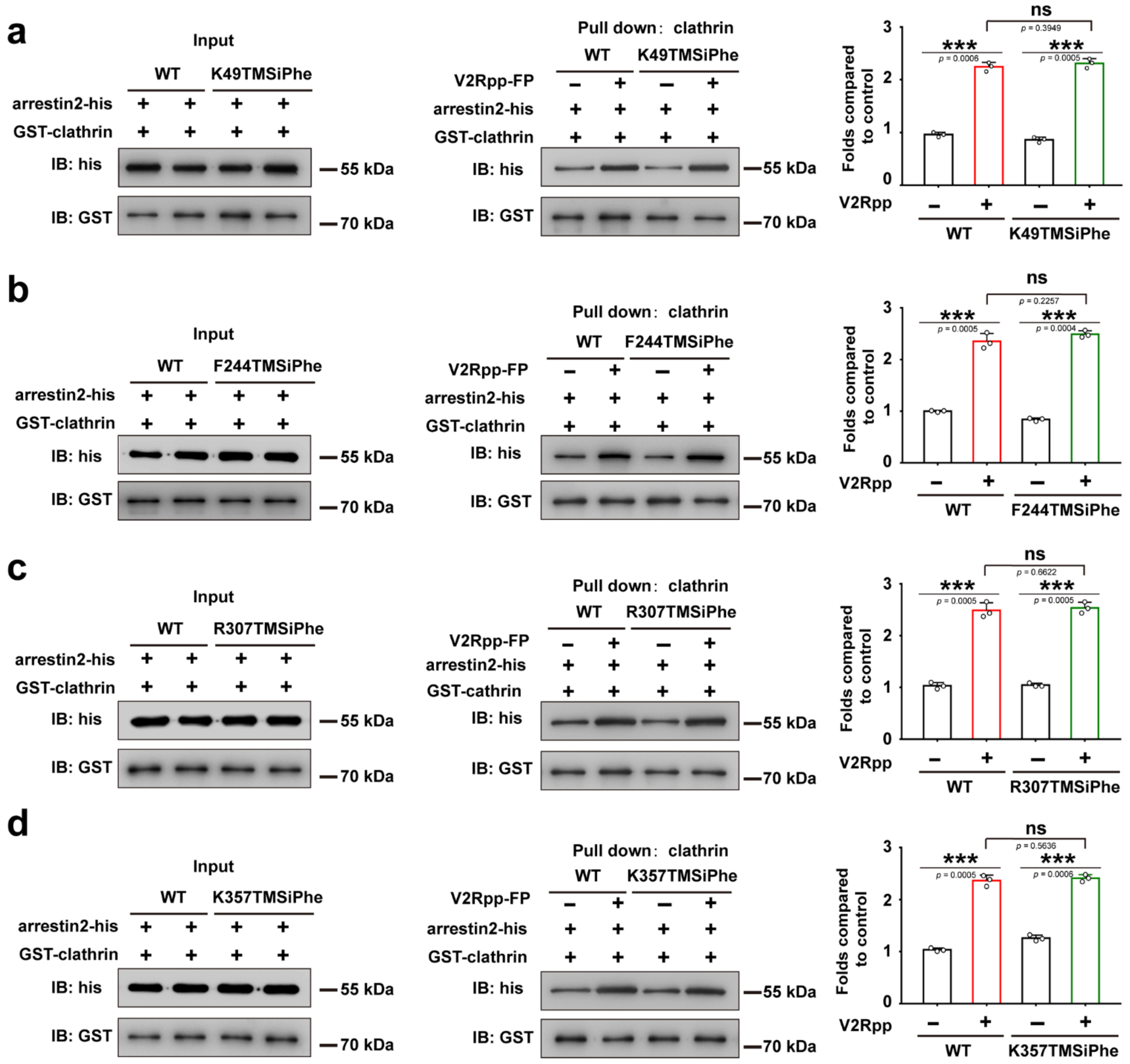

Supplementary Fig. 12. Functional analysis of arrestin2 with incorporated TMSiPhe on V2Rpp-FP promoted arrestin/clathrin complex formation.

a-d). Wild type (0.1 μM), (a) K49TMSiPhe, (b) F244TMSiPhe, (c) R307TMSiPhe, and (d) K357TMSiPhe mutants of arrestin2 were mixed with 0.5 μM V2Rpp-FP in binding buffer (20 mM Tris-HCl, pH = 7.5, 150 mM NaCl) at 25 °C for 30 min. After incubation, 1 μM GST-clathrin was added and incubated for another 1 h. The complexes were pulled down using GST beads, and the amount of arrestin bound to clathrin was determined by using anti-His antibody. The results suggest that arrestin2 with incorporated TMSiPhe exhibited functional integrity in vitro. The western blot signals were quantified and are shown as columns in the right panel, +: with V2Rpp-FP; -: without V2Rpp-FP. Statistics were determined by

one-way ANOVA with Tukey's test. ***, $p<0.001$ (V2Rpp-FP stimulation was compared with control vehicles). Values are the mean ± SEM of three independent experiments (n=3) for the wild type (WT) and mutants. Full blot images of Supplementary Fig. 12 were shown in Supplementary Fig. 18 and Source Data file.

Supplementary Fig. 13

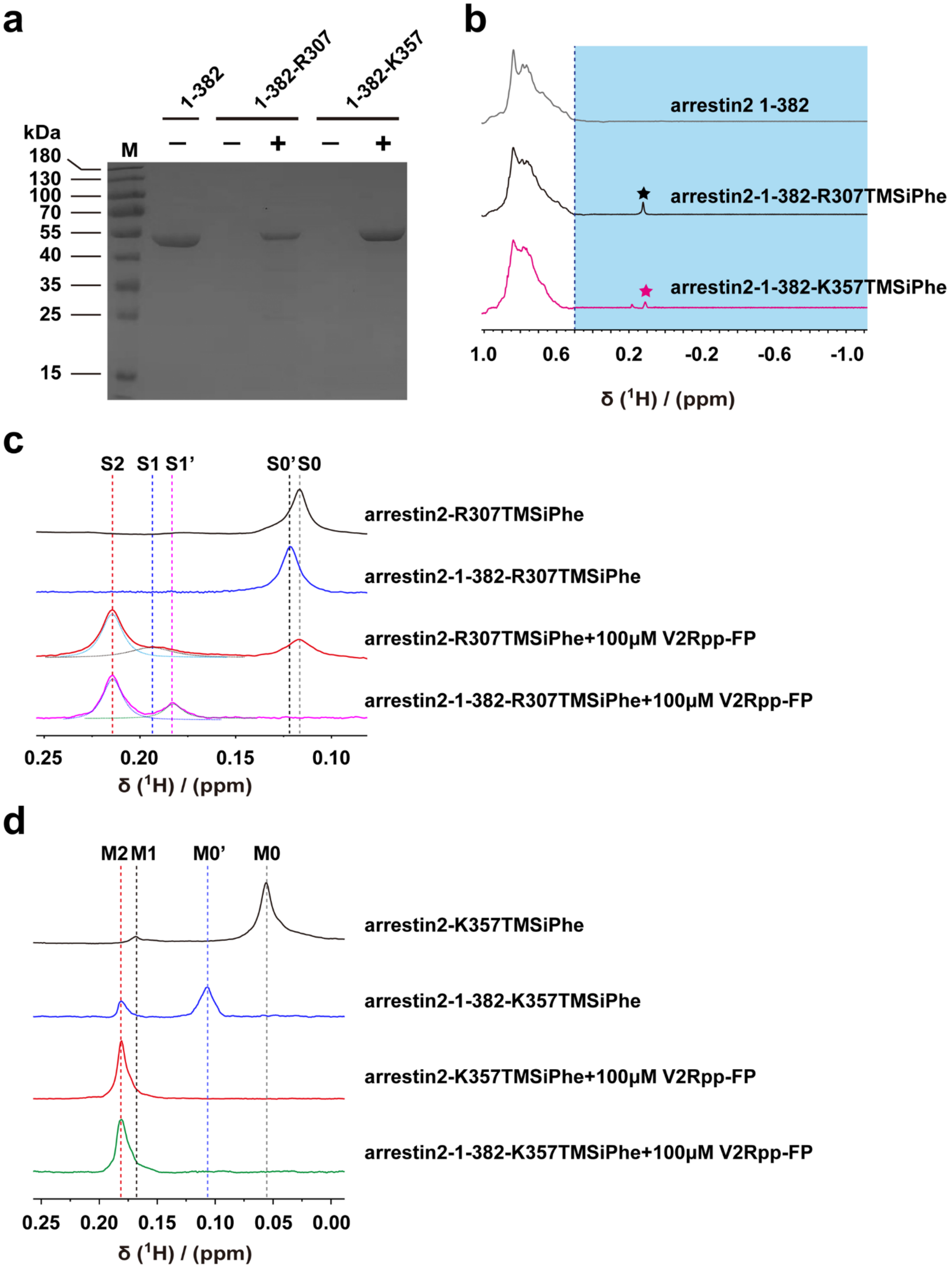

Supplementary Fig. 13. Incorporation of TMSiPhe at R307 and K357 motifs of 1-382 truncated arrestin2.

a). Coomassie-stained gel was used to analyze the purified wild-type 1-382 truncated arrestin2 or R307 and K357 mutants with TMSiPhe incorporation. This experiment was repeated three times (n=3) independently with similar results. +: Presence of 1 mM TMSiPhe in the culture medium; -: Absence of 1 mM TMSiPhe in the culture medium.

b). 1D ^{1}H NMR spectra of arrestin2 labelled at position R307 and K357. The spectra were recorded with 10 μM TMSiPhe-incorporated arrestin2, and the total recording time per spectrum was 15 min. The chemical shift for analysis was less than 0.55 ppm. The pentagrams: the position of NMR signal peak for the arrestin2 1-382 inserted TMSiPhe at different sites. Black: R307; pink: K357.

c). 1D ^{1}H NMR spectra of full-length and 1-382 truncated arrestin2-R307TMSiPhe in response to incubation with V2Rpp-FP or control vehicles. The corresponding NMR shifts at 0,116 ppm, 0.122 ppm, 0.195 ppm, 0.184 ppm and 0.219 ppm are designated S0, S0', S1, S1' and S2, respectively.

d). 1D ^{1}H NMR spectra of full-length and 1-382 truncated arrestin2-K357TMSiPhe in response to incubation with V2Rpp-FP or control vehicles. The corresponding NMR shifts at 0.055 ppm, 0.110 ppm 0.170 ppm and 0.180 ppm are designated M0, M0', M1 and M2 respectively.

Supplementary Fig. 14

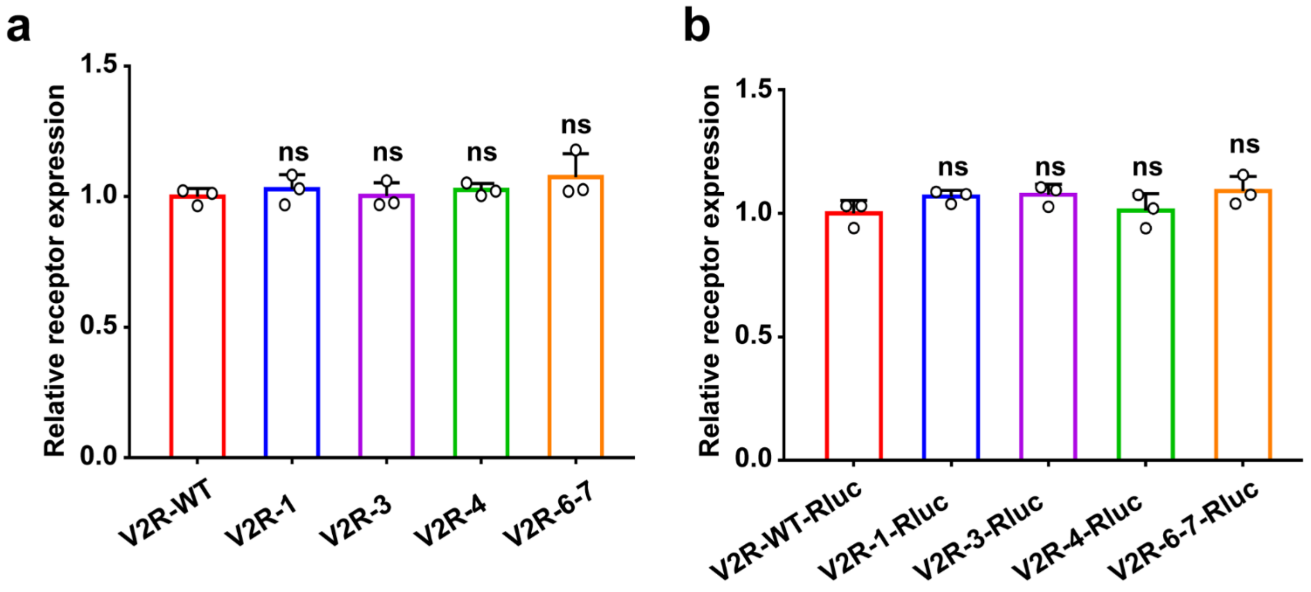

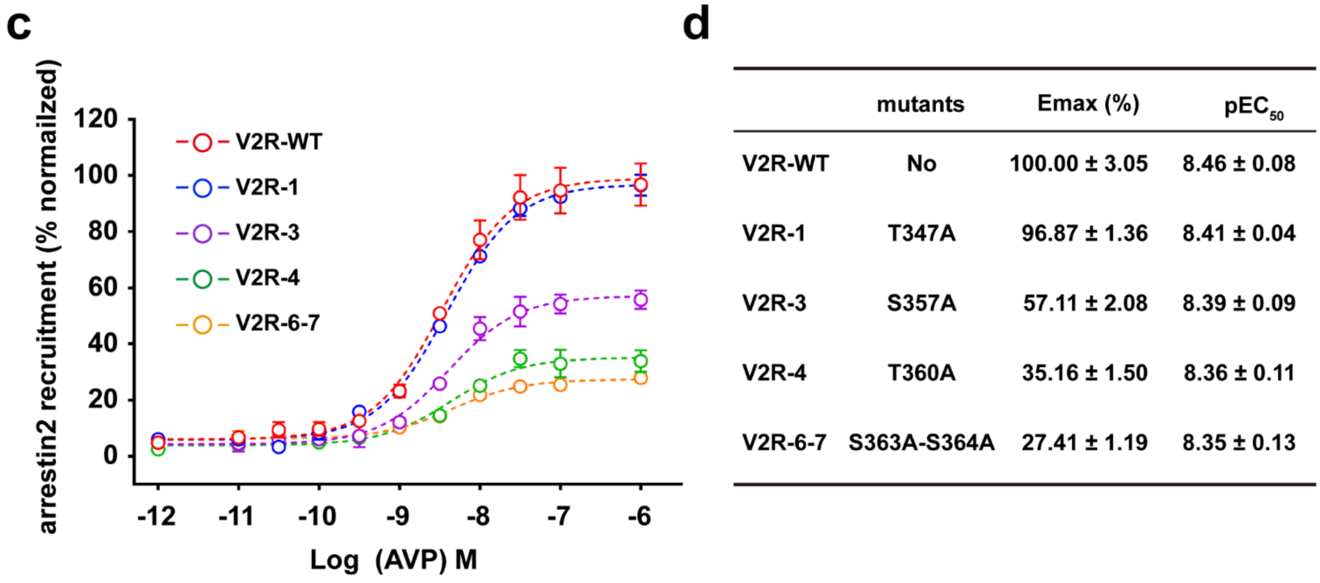

d

	mutants	Emax (%)	pEC_{50}
V2R-WT	No	100.00 ± 3.05	8.46 ± 0.08
V2R-1	T347A	96.87 ± 1.36	8.41 ± 0.04
V2R-3	S357A	57.11 ± 2.08	8.39 ± 0.09
V2R-4	T360A	35.16 ± 1.50	8.36 ± 0.11
V2R-6-7	S363A-S364A	27.41 ± 1.19	8.35 ± 0.13

Supplementary Fig. 14. Surface expression and arrestin2 recruitment toward different V2R phosphor-deficient mutants.

a-b). Elisa experiments to assay the expression levels of the wild type and indicated mutants of V2R or V2R-Rluc in HEK293 cells. Data from three independent experiments (n=3) are presented as mean ± SD. Statistical differences between WT and mutations were determined by two-sided one-way ANOVA with Tukey test. “ns”: the mutants showed no significant difference compared to V2R-WT.

c). The vasopressin-induced arrestin2 recruitment to different V2R phospho-deficient mutants were compared with V2R-WT, examined by BRET assay in a concentration dependent manner. n = 3 biologically independent replicates were examined for three independent experiments. Data (means ± SEM) of each mutant were normalized to

maximal response of V2R-WT.

d). Statistical analysis of the Emax and pEC50 of vasopressin induced arrestin2 recruitment to V2R WT or V2R mutants.

Supplementary Fig. 15

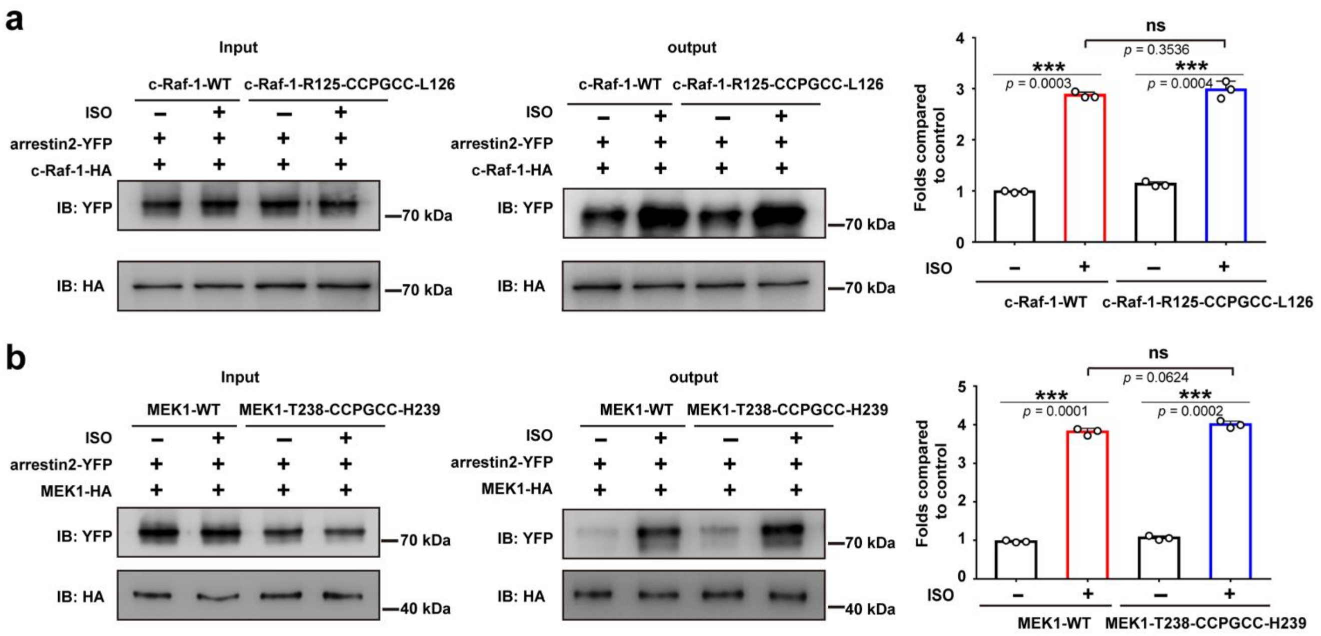

Supplementary Fig. 15. Functional analysis of c-Raf-1/MEK1 FlAsH biosensor on isoproterenol (ISO) induced arrestin2 complex formation.

HEK293 cells were co-transfected with plasmids encoded the YFP-arrestin2 and HA-c-Raf-1-WT or HA-c-Raf-1-CCPGCC (a); HA-MEK1-WT or HA-MEK1-CCPGCC (b). Forty hours after transfection, the cells were starved for 8 h and stimulated with 10 μM isoproterenol (ISO) for 15 min. HA-MEK1/HA-c-Raf-1 was immunoprecipitated by HA-antibody-conjugated agarose, and the arrestin2 were detected by western blotting using specific YFP antibodies. The results suggest that that these FlAsH biosensors of c-Raf-1 or MEK1 are able to recognize active arrestin. Full blot images of Supplementary Fig. 15 were shown in Supplementary Fig. 19 and Source Data file. The western blot signals were quantified and are shown as columns in the right panel, +: with ISO; -: without ISO. Statistics were determined by one-way ANOVA with Tukey's test. ***, $p<0.001$ (ISO stimulation was compared with control vehicles). Values are the mean ± SEM of three independent experiments (n=3) for the wild type (WT) and mutants.

Supplementary Fig. 16

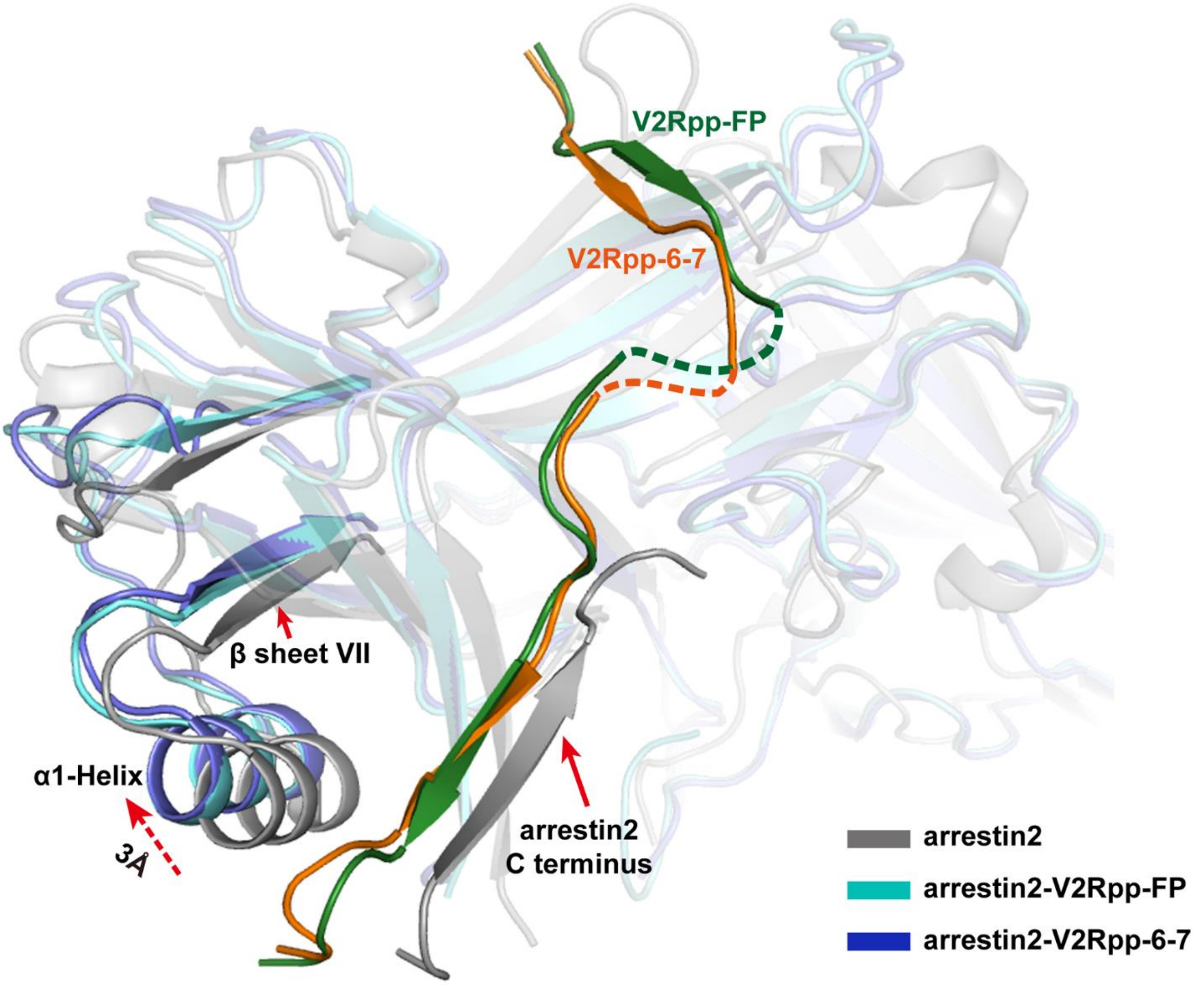

Supplementary Fig. 16. Conformational change of the α-Helix and β sheet VII-α1 after arrestin activation in response to interaction with the V2Rpp-FP or the V2Rpp-6-7.

Binding of the V2Rpp-FP and the V2Rpp-6-7 which replaced the C-tail of arrestin both caused an approximately 3 Å shift of the N-terminal α-helix and the reorganization of the β sheet VII-α1 bulge.

Supplementary Fig. 17

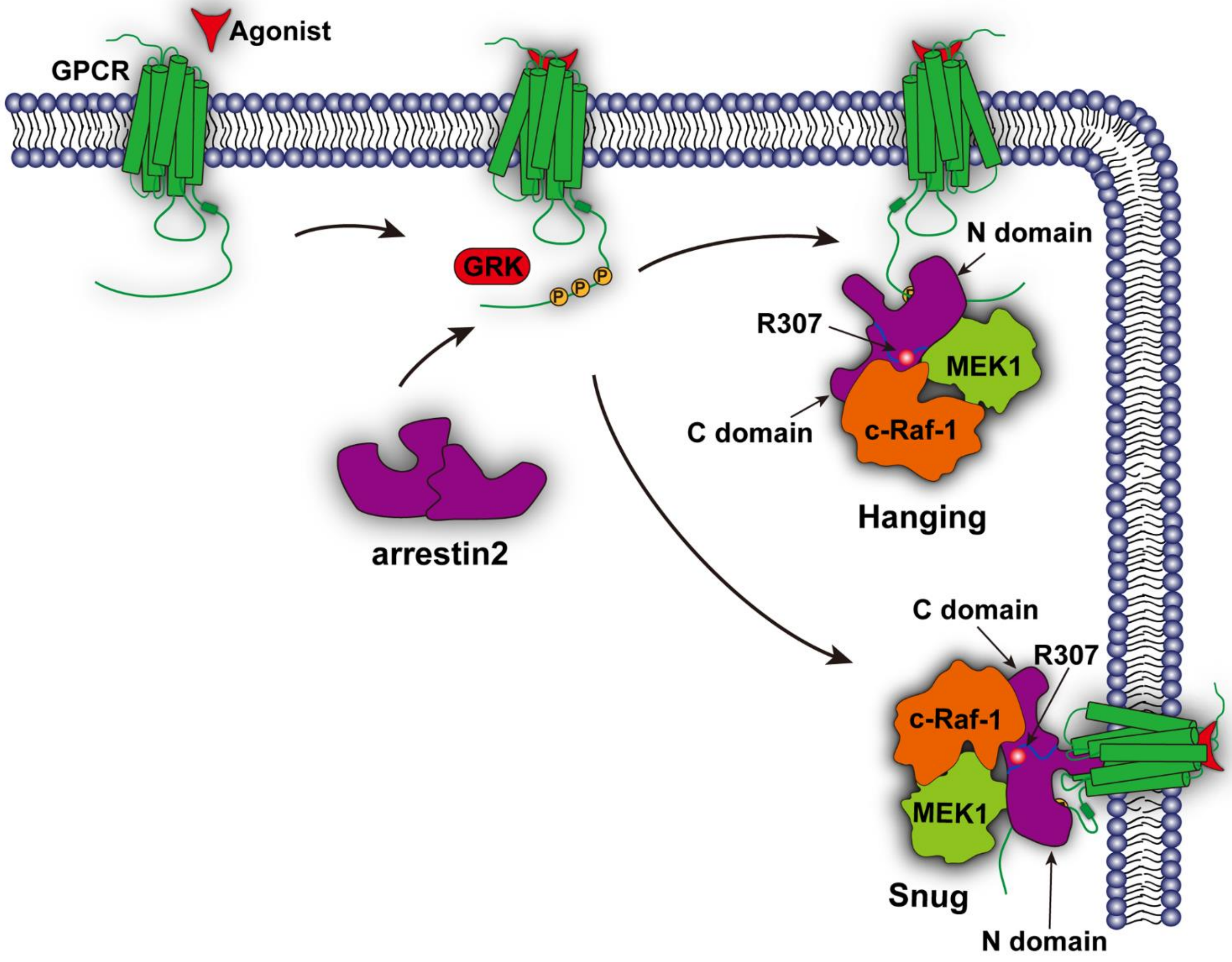

Supplementary Fig. 17. Schematic representation of interaction modes of GPCR-arrestin2-MEK1/c-Raf-1 complexes, in snug or hanging conformations. GPCRs phosphorylated by GRKs enabled the recruitment of arrestin through the phosphorylated carboxyl termini of receptors to form either "Hanging" or "Snug" state, which subsequently recruit MEK1 or c-Raf-1 after receptor activation. The red ball: R307 site in arrestin2; the yellow circles: phosphorylation.

Supplementary Fig. 18

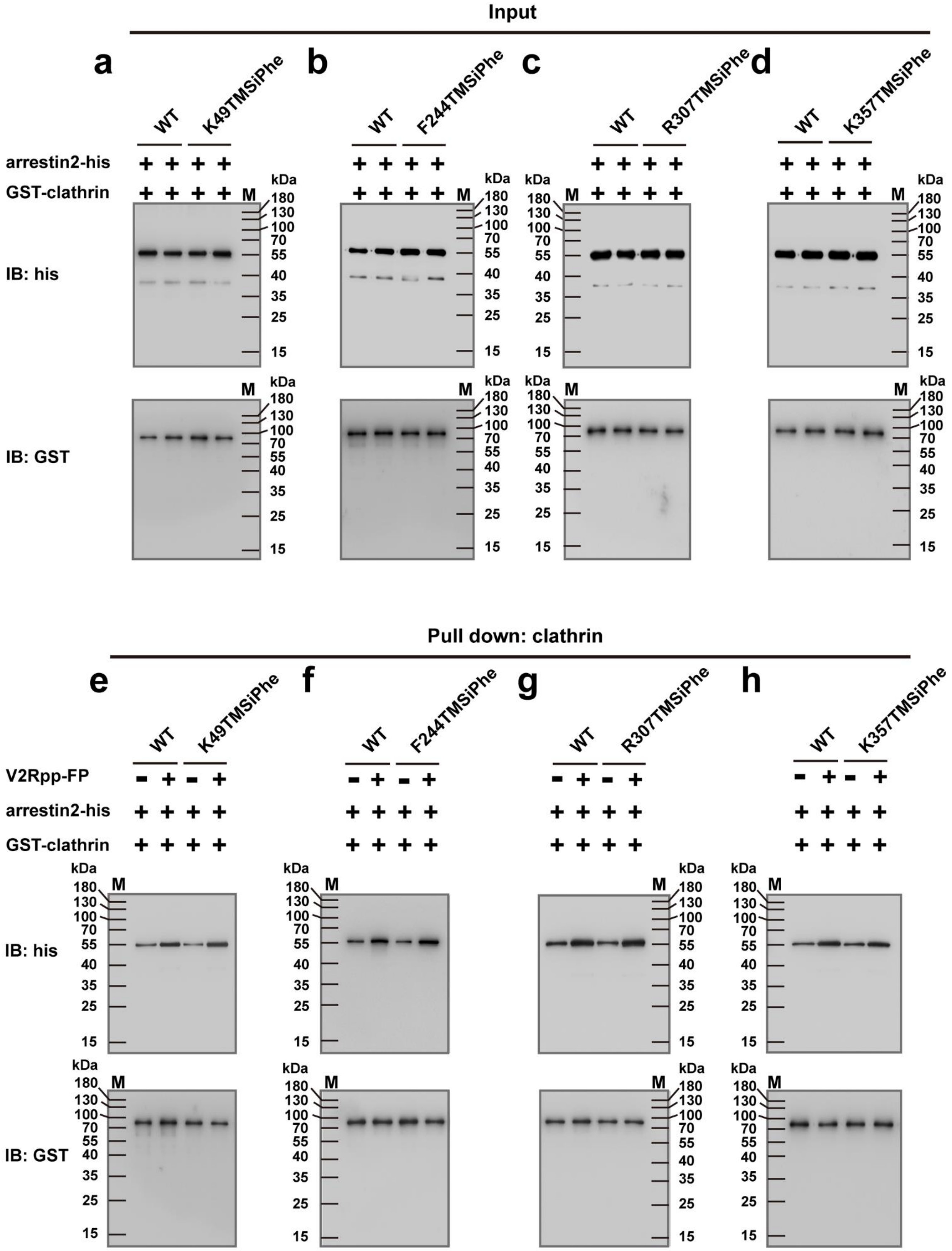

Supplementary Fig. 18. Full blot images of Supplementary Fig. 12.

Supplementary Fig. 19

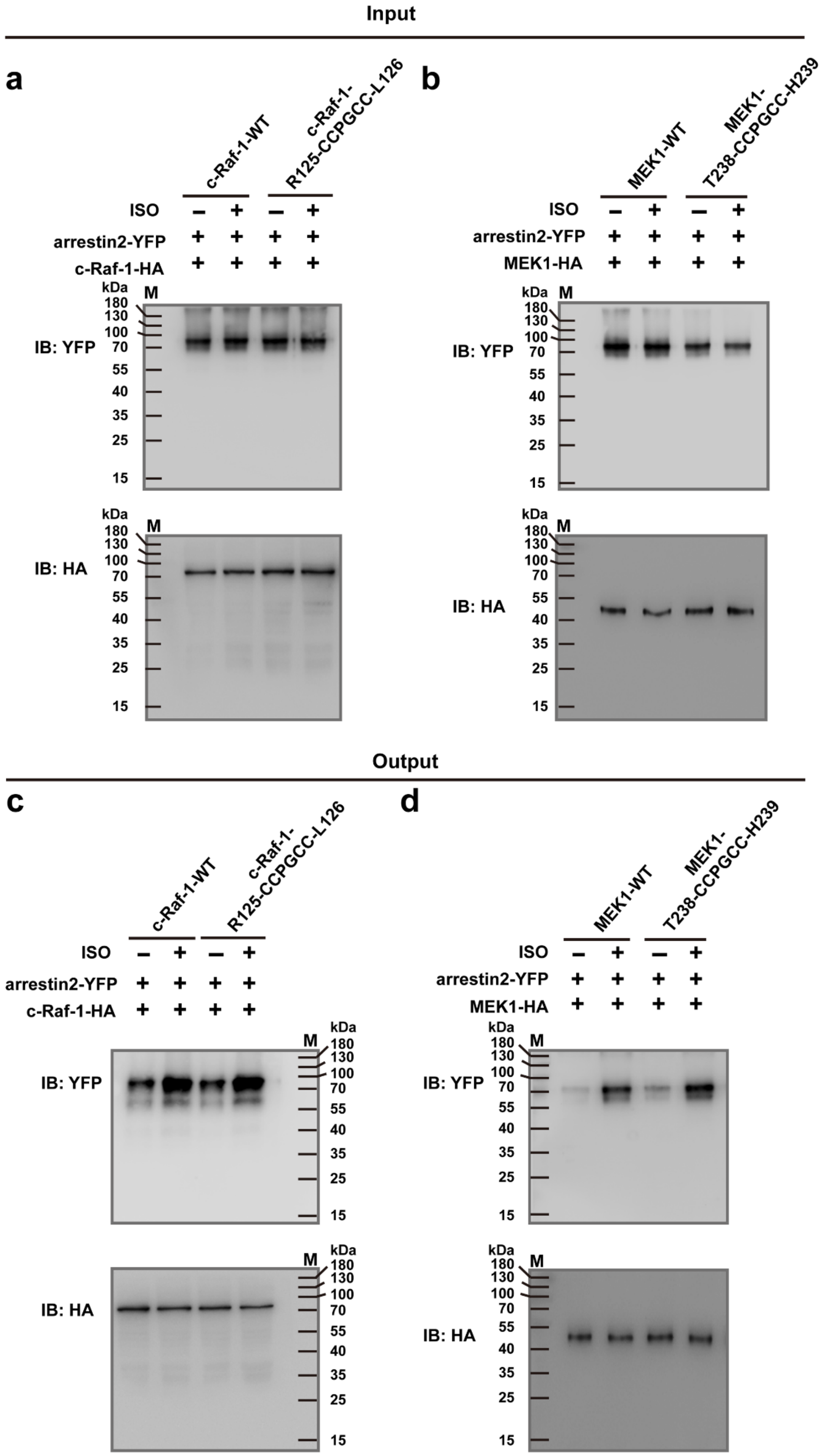

Supplementary Fig. 19. Full blot images of Supplementary Fig. 15.

Supplementary Table 1. Data collection and refinement statistics.

	arrestin2-V2Rpp-1-Fab30	arrestin2-V2Rpp-3-Fab30	arrestin2-V2Rpp-4-Fab30	arrestin2-V2Rpp-6-7-Fab30
**Data Collection**				
Space group	$I2_12_12_1$	$I2_12_12_1$	$I2_12_12_1$	$I2_12_12_1$
Cell Dimensions				
a. b. c (Å)	116.17, 121.54, 144.64	116.14, 122.65, 144.06	116.90, 121.14, 144.50	115.48, 116.88, 143.90
α, β, γ (deg)	90, 90, 90	90, 90, 90	90, 90, 90	90, 90, 90
Resolution (Å)	39.01-3.17 (3.28-3.17) *	44.72-2.49 (2.57-2.49) *	46.42-2.54 (2.63-2.54) *	45.36-3.28 (3.40-3.28) *
R_{sym} or R_{merge}	0.082 (1.340)	0.061 (0.856)	0.076 (1.822)	0.094 (0.929)
I / σ	9.8 (1.0)	20.5 (2.2)	14.3 (1.1)	14.0 (2.5)
Completeness (%)	99.8 (99.9)	99.5 (98.8)	99.9 (99.8)	99.5 (99.0)
Redundancy	5.6 (6.1)	7.3 (7.2)	6.6 (6.8)	6.5 (6.9)
**Refinement**				
Resolution (Å)	39.01-3.17 (3.28-3.17)	44.72-2.49 (2.57-2.49)	46.42-2.54 (2.63-2.54)	45.36-3.28 (3.40-3.28)
No. reflections	17638 (1735)	36384 (3573)	34241 (3379)	15094 (1478)
R_{work} / R_{free}	0.254/0.288	0.229/0.285	0.234/0.281	0.232/0.290
No. atoms	5742	5978	6017	5913
Protein	5742	5915	6007	5913
Ligand/ion	—	6	10	—
No. atoms	—	57	—	—
B-factors($Å^2$)	106.43	87.62	84.25	113.16
Protein($Å^2$)	106.43	87.77	84.26	113.16
Ligand/ion($Å^2$)	—	87.31	—	—
R.M.S. deviations				
Bond lengths (Å)	0.012	0.009	0.009	0.010
Bond angles (deg)	1.36	1.13	1.12	1.25

*** Number of xtals for each structure should be noted in footnote. *Highest-resolution shell is shown in parentheses.**

[AU: Equations defining various R values are standard and hence are no longer defined in the footnotes.]

[AU: Ramachandran statistics should be in methods section at the end of the refinement sub-section.]

[AU: Wavelength of data collection, temperature, beamline should all be in methods section.]

Supplementary Table 2. The TMSiPhe incorporation sites in arrestin2 and their proposed functions reported in the literature.

Position	Description	Key literature
K49	Nuclear localization sequence (NLS) region of arrestin2.	Peterson et al., *Pharmacol Rev.* 2017
F87	Polyproline Ⅰ region of arrestin2 mediates SH3 protein coupling.	Yang et al., *Nat. Chem. Biol.* 2018
S193	Site region that endocytosis is mediated by the interaction of arrestin and clathrin.	Eichel et al., *Nat.* 2018;
F244	Site region that related to arrestin coupling with receptor.	Latorraca et al., *Nat.* 2018;
R307	Site region that exhibits arrestin coupling with c-Raf-1.	Coffa et al., *Biochem.* 2011
L334	Site region that endocytosis is mediated by the interaction of arrestin and clathrin.	Kang et al., *JBC*. 2009
K357	Arrestin C tail region interaction with MEK1.	Cassier et al., *elife. 2017*

Supplementary Table 3: The interactions between site V1 of arrestin2 and V2Rpp-1 compared to V2Rpp-FP.

Site	Interaction	arrestin2	V2Rpp-1	Distance (Å)	V2Rpp-FP	Distance (Å)
V1	H-bond (≤3.5 Å)	Y63	T347	—	pT347	2.41
		R65		—		3.11
		K77		—		2.71
	Anion-π interaction (≤6.0 Å)	F75		—		4.59
	Polar interaction (≤4.0 Å)	R76 (main chain)		3.87		—
	Hydrophobic interaction (≤4.0 Å)	F75		3.95		3.72

[a]Data given as "-" mean that distance was beyond the critical value to form relevant interactions, e.g., H-bond interactions were not counted in case of distance > 3.5 Å; Anion-π interactions were not counted in case of distance > 6.0 Å; Polar interactions were not counted in case ofdistance > 4.0 Å. [b]Data given as "/" mean that residue could not be unambiguously assigned by electron density.

Supplementary Table 4

Sample		S0 (0.116 ppm)			S1 (0.195 ppm)			S2 (0.219 ppm)		
		Peak areas (x10³)	Resdiual error (x10²)	S/N	Peak areas (x10³)	Resdiual error (x10²)	S/N	Peak areas (x10³)	Resdiual error (x10²)	S/N
R307TMSiPhe	---	6.9	2.5	36.7	---	---	---	---	---	---
	V2Rpp-FP	2.1	0.8	12.5	3.6	0.7	5.6	6.5	0.3	31.5
	V2Rpp-1	1.9	2.6	24.9	4.9	2.2	31.6	4.0	1.7	31.3
	V2Rpp-3	1.7	1.5	12.5	4.4	1.1	19.6	5.1	1.0	28.9
	V2Rpp-4	7.5	0.5	40.5	---	---	---	---	---	---
	V2Rpp-6-7	7.3	1.0	48.2	---	---	---	---	---	---

Supplementary Table 4. ^{1}H-NMR characteristic signal peak position and area analysis of the complexes of R307TMSiPhe arrestin2/V2Rpp-FP with different phospho-peptides.

The fitting region of the S0, S1 and S2 were at 0.070~0.150 ppm, 0.160~0.210ppm, 0.210~0.240 ppm respectively, and then the area of the main peaks and corresponding residual error were obtained. Signal-to-noise ratio was measured using 'sinocal' routine within Topspin 4.0 (Bruker Biospin, Billerica MA), on the TMSiPhe signal of R307TMSiPhe arrestin2 at 0.116ppm, 0.195ppm or 0.219 ppm, using 2 ppm noise regions (centered around 11

ppm) for SNR calculations.

Supplementary Table 5

Sample		S0' (0.122 ppm)			S1' (0.184 ppm)			S2 (0.219 ppm)		
		Peak areas (x10^3)	Resdiual error (x10^2)	S/N	Peak areas (x10^3)	Resdiual error (x10^2)	S/N	Peak areas (x10^3)	Resdiual error (x10^2)	S/N
R307TMSiPhe 1-382	---	8.2	1.5	263.0	---	---	---	---	---	---
	V2Rpp-FP	---	---	---	2.0	0.5	10.7	5.0	1.6	34.1

Supplementary Table 5. ^{1}H-NMR characteristic signal peak position and area analysis of the complexes of R307TMSiPhe 1-382 truncated arrestin2 with V2Rpp-FP.

The fitting region of the S0', S1' and S2 were at 0.080~0.160 ppm, 0.150~0.200 ppm, 0.200~0.250 ppm respectively, and then the area of the main peaks and corresponding residual error were obtained. Signal-to-noise ratio was measured using 'sinocal' routine within Topspin 4.0 (Bruker Biospin, Billerica MA), on the TMSiPhe signal of K357TMSiPhe 1-382 truncated arrestin2 at 0.122 ppm, 0.184 ppm or 0.219 ppm, using 2 ppm noise regions (centered around 11 ppm) for SNR calculations.

Supplementary Table 6：The interactions between site V3 of arrestin2 and V2Rpp-3 compared to V2Rpp-FP.

Site	Interaction	arrestin2	V2Rpp-3	Distance (Å)	V2Rpp-FP	Distance (Å)
V3	**H-bond (≤3.5 Å)**	K11	S357	—	pS357	2.86
	**Polar interaction (≤4.0 Å)**	K11		3.70		—
		K160		—		3.74
	**Charge-Charge (≤6.0 Å)**	K138		—		5.20
		R165		—		4.02

[a]Data given as "-" mean that distance was beyond the critical value to form relevant interactions, e.g., H-bond interactions were not counted in case of distance > 3.5 Å; Anion-π interactions were not counted in case of distance > 6.0 Å; Polar interactions were not counted in case of distance > 4.0 Å. Charge-Charge interactions were not counted in case of distance > 6.0 Å.

Supplementary Table 7

Sample		R0 (0.091 ppm)			R1 (0.026 ppm)			R2 (0.019 ppm)		
		Peak areas (x10^3)	Resdiual error (x10^2)	S/N	Peak areas (x10^3)	Resdiual error (x10^2)	S/N	Peak areas (x10^3)	Resdiual error (x10^2)	S/N
F244TMSiPhe	---	23.6	12.7	17.7	2.5	58.9	3.0	---	---	---
	V2Rpp-FP	---	---	---	19.8	7.2	24.6	---	---	---
	V2Rpp-3	1.5	5.2	7.3	---	---	---	19.3	7.6	25.7

Supplementary Table 7. ^{1}H-NMR characteristic signal peak position and area analysis of the complexes of F244TMSiPhe arrestin2 with V2Rpp-FP and V2Rpp-3.

The fitting region of the R0, R1 and R2 were at 0.060~0.120 ppm, 0.000~0.070 ppm, -0.030~0.060 ppm respectively, and then the area of the main peaks and corresponding residual error were obtained. Signal-to-noise ratio was measured using 'sinocal' routine within Topspin 4.0 (Bruker Biospin, Billerica MA), on the TMSiPhe signal of F244TMSiPhe arrestin2 at 0.091ppm, 0.026ppm or 0.019 ppm, using 2 ppm noise regions (centered around 11 ppm) for SNR calculations.

Supplementary Table 8

Sample		M0 (0.055 ppm)			M1 (0.170 ppm)			M2 (0.180 ppm)			M3 (0.026ppm)		
		Peak areas (x10^3)	Resdiual error (x10^2)	S/N	Peak areas (x10^3)	Resdiual error (x10^2)	S/N	Peak areas (x10^3)	Resdiual error (x10^2)	S/N	Peak areas (x10^3)	Resdiual error (x10^2)	S/N
K357TMSiPhe	---	41.6	25.2	135.8	2.4	11.8	13.4	---	---	---	---	---	---
	V2Rpp-FP	---	---	---	---	---	---	34.7	34.6	113.8	---	---	---
	V2Rpp-1	---	---	---	---	---	---	39.2	18.1	135.4	---	---	---
	V2Rpp-3	11.6	2.9	21.8	2.6	6.2	13.2	2.5	3.3	11.9	16.6	4.7	22.6
	V2Rpp-4	24.0	12.0	35.5	2.4	1.5	6.0	---	---	---	7.4	0.8	21.2
	V2Rpp-6-7	21.6	17.6	23.0	2.8	2.5	4.1	---	---	---	8.3	4.6	15.3

Supplementary Table 8. ^{1}H-NMR characteristic signal peak position and area analysis of the complexes of K357TMSiPhe arrestin2/V2Rpp-FP with different phospho-peptides.

The fitting region of the M0, M1, M2 and M3 were at 0.000~0.100 ppm, 0.160~0.185 ppm, 0.140~0.200 ppm, -0.040~0.040 ppm respectively, and then the area of the main peaks and corresponding residual error were obtained. Signal-to-noise ratio was measured using 'sinocal' routine within Topspin 4.0 (Bruker Biospin, Billerica MA), on the TMSiPhe signal of K357TMSiPhe arrestin2 at 0.055ppm, 0.170ppm, 0.180 ppm or 0.026 ppm, using 2 ppm noise regions (centered around 11 ppm) for SNR calculations.

Supplementary Table 9

Sample		M0’ (0.110 ppm)			M2 (0.180 ppm)		
		Peak areas (x10^3)	Resdiual error (x10^2)	S/N	Peak areas (x10^3)	Resdiual error (x10^2)	S/N
K357TMSiPhe 1-382	---	2.3	0.2	9.2	0.7	0.2	17.8
	V2Rpp-FP	---	---	---	3.3	0.3	45.3

Supplementary Table 9. ^{1}H-NMR characteristic signal peak position and area analysis of the complexes of K357TMSiPhe 1-382 truncated arrestin2 with V2Rpp-FP.

The fitting region of the M0’ and M2 were at 0.080~0.140 ppm, 0.150~0.210 ppm respectively, and then the area of the main peaks and corresponding residual error were obtained. Signal-to-noise ratio was measured using ‘sinocal’ routine within Topspin 4.0 (Bruker Biospin, Billerica MA), on the TMSiPhe signal of R307TMSiPhe 1-382 truncated arrestin2 at 0.110 ppm or 0.180 ppm, using 2 ppm noise regions (centered around 11 ppm) for SNR calculations.

Supplementary Table 10：The interactions between site V6/V7 of arrestin2 and V2Rpp-6-7 compared to V2Rpp-FP.

Site	Interaction	arrestin2	V2Rpp-6-7	Distance (Å)	V2Rpp-FP	Distance (Å)
V6 / V7	H-bond (≤3.5 Å)	R7	S363	3.45	pS363	—
		V8 (main chain)		3.00		—
		K10		3.38		2.47
		Y21		—		3.21
		K107		—		3.28
	Polar interaction (≤4.0 Å)	R7		—		3.80
		V8 (main chain)		—		3.52
	H-bond (≤3.5 Å)	T6 (main chain)	S364	3.34	pS364	—
		K107		—		2.27
	Polar interaction (≤4.0 Å)	R7		3.61		—
	Charge-Charge (≤6.0 Å)	R7		—		4.86
		K107		4.49		—

[a]Data given as "-" mean that distance was beyond the critical value to form relevant interactions, e.g., H-bond interactions were not counted in case of distance > 3.5 Å; Anion-π interactions were not counted in case of distance > 6.0 Å; Polar interactions were not counted in case of distance > 4.0 Å. Charge-Charge interactions were not counted in case of distance > 6.0 Å.

Supplementary Table 11

Sample		N0 (0.135 ppm)			N1 (0.149 ppm)		
		Peak areas (x10³)	Resdiual error (x10²)	S/N	Peak areas (x10³)	Resdiual error (x10²)	S/N
K49TMSiPhe	---	19.8	2.3	45.5	---	---	---
	V2Rpp-FP	---	---	---	18.0	3.7	102.4
	V2Rpp-1	---	---	---	17.0	4.5	97.2
	V2Rpp-3	7.7	13.6	36.7	13.8	7.2	47.8
	V2Rpp-4	17.7	28.4	30.4	---	---	---
	V2Rpp-6-7	16.7	24.9	29.2	---	---	---

Supplementary Table 11. ^{1}H-NMR characteristic signal peak position and area analysis of the complexes of K49TMSiPhe arrestin2/V2Rpp with different phospho-peptides.

The fitting region of the N1and N2 were at 0.100~0.150 ppm, 0.120~0.170 ppm respectively, and then the area of the main peaks and corresponding residual error were obtained. Signal-to-noise ratio was measured using 'sinocal' routine within Topspin 4.0 (Bruker Biospin, Billerica MA), on the TMSiPhe signal of K49TMSiPhe arrestin2 at 0.135 ppm or 0.149 ppm, using 2 ppm noise regions (centered around 11 ppm) for SNR calculations.

Supplementary Table 12: The interactions between site V3’4’ of arrestin2 and VRpp-4 compared to V2Rpp-FP.

Site	Interaction	arrestin2	V2Rpp-4	Distance (Å)	V2Rpp-FP	Distance (Å)
V3’4’	**H-bond (≤3.5 Å)**	K11	pS357	—	pS357	2.86
	**Polar interaction (≤4.0 Å)**	K160		—		3.74
	**Charge-Charge (≤6.0 Å)**	K138		—		5.20
		R165		—		4.02
	**H-bond (≤3.5 Å)**	K11	pT359	2.85	pT359	—
	**Charge-Charge (≤6.0 Å)**	R25		4.97		—
	**H-bond (≤3.5 Å)**	K11	T360	—	pT360	2.65
		R25		—		3.45
		K294		—		2.94
	**Polar interaction (≤4.0 Å)**	R25		3.85		—
	**Charge-Charge (≤6.0 Å)**	K11		4.51		—

[a]Data given as “-” mean that distance was beyond the critical value to form relevant interactions, e.g., H-bond interactions were not counted in case of distance > 3.5 Å; Anion-π interactions were not counted in case of distance > 6.0 Å; Polar interactions were not counted in case of distance > 4.0 Å. Charge-Charge interactions were not counted in case of distance > 6.0 Å.

Supplementary Table 13: A complete list of all primers used in our study.

Name	Sequence
arrestin2-K49TAG-F	TATCTCTAGGAGAGGAGAGTCTATGTG
arrestin2-K49TAG-R	CCTCTCCTAGAGATACTCCGGATCCAC
arrestin2-F244TAG-F	TGTCTGTAGAACACAGCCCAGTACAAG
arrestin2-F244TAG-R	TGTGTTCTACAGACAGATGTCTGCATA
arrestin2-R307TAG-F	CTGTTGTAGGAAGGAGCCAACCGGGAG
arrestin2-R307TAG-R	TCCTTCCTACAACAGGGTGCTGGAGGC
arrestin2-K357TAG-F	AAGCCCTAGGAGGAACCCCCACACCGG
arrestin2-K357TAG-R	TTCCTCCTAGGGCTTGGGGTGCATTAG
arrestin2-1-382-F	GACACCAACCTCGAGCACCACCACCACCACCAC
arrestin2-1-382-R	CTCGAGGTTGGTGTCAAGTTCTATGAGATTGGT

pcDNA3.1-V2R-T347A-F	GGACGCGCGCCACCCAGCCTGGGTCCCCAA
pcDNA3.1-V2R-T347A-R	GGGTGGCGCGCGTCCCCGGGCACAGCAGAG
pcDNA3.1-V2R-S357A-F	GATGAGGCGTGCACCACCGCCAGCTCCTCC
pcDNA3.1-V2R-S357A-R	GGTGCACGCCTCATCTTGGGGACCCAGGCT
pcDNA3.1-V2R-T360A-F	TGCACCGCGGCCAGCTCCTCCCTGGCCAAG
pcDNA3.1-V2R-T360A-R	GCTGGCCGCGGTGCAGGACTCATCTTGGGG
pcDNA3.1-V2R-S362A-S363A-F	GCCAGCGCGGCGCTGGCCAAGGACACTTCATCGTGA
pcDNA3.1-V2R-S362A-S363A-R	GGCCAGCGCCGCGCTGGCGGTGGTGCAGGACTC
pcDNA3.1-arrestin2-R-vector-F	GCAAGCTTGATATCCTGCGGCGACAAAGGGACACGAGTG TTC
pcDNA3.1-arrestin2-R-vector-R	GTACACCTTGCTGGTCATGGTCCGATCTGCAGAATTCCAG CA
gene of Rluc-F	ATGACCAGCAAGGTGTACGACCCCGAGCAG
gene of Rluc-R	GGATCCGGTACCGCAAGCTTGATATCCTGC
pcDNA3.1-c-Raf-1-R125-CCPGCC-L126-F	TGCTGCCCGGGATGCTGCTTAGATTGGAATACTGATGCTG CG
pcDNA3.1-c-Raf-1-R125-CCPGCC-L126-R	GCATCCCGGGCAGCAGCGTGCTTTTTACCTTTGTGTTCG TG
pcDNA3.1-MEK1-T238-CCPGCC-H239-F	TGCTGCCCGGGATGCTGCCATTACTCTGTGCAGTCAGAC ATC
pcDNA3.1-MEK1-T238-CCPGCC-H239-R	GCATCCCGGGCAGCAAGTCCCCTGGAGTCTTTCTGGCGA CAT
pcDNA3.1-V2R-R-vector-F	GCTTGCGGTACCGGATCCTGATCTAGAGGATCCCGGGTG GCA
pcDNA3.1-V2R-R-vector-R	GTACACCTTGCTGGTCATCGATGAAGTGTCCTTGGCCAG
pcDNA3.1-arrestin2-Y-vector-F	CTCGTCCATGCCGAGAGTTAGTCTAGAGGGCCCTATTCT
pcDNA3.1-arrestin2-Y-vector-R	CTCGCCCTTGCTCACCATTCTGTTGTTGAGGTGTGGAGA GCC
gene of YFP-F	ATGGTGAGCAAGGGCGAGGAGCTGTTC
gene of YFP-R	CTTGTACAGCTCGTCCATGCCGAGAGT

Supplementary References：

1 Liu, Q. *et al.* DeSiphering receptor core-induced and ligand-dependent conformational changes in arrestin via genetic encoded trimethylsilyl ^{1}H-NMR probe. *Nat Commun* **11**, 4857, (2020).

2 Shukla, A. K. *et al.* Structure of active β-arrestin-1 bound to a G-protein-coupled receptor phosphopeptide. *Nature* **497**, 137-141, (2013).

3 Nguyen, A. H. *et al.* Structure of an endosomal signaling GPCR-G protein-β-arrestin megacomplex. *Nat*

Struct Mol Biol **26**, 1123-1131, (2019).

4 Huang, S. M. *et al.* Genetically Encoded Fluorescent Amino Acid for Monitoring Protein Interactions through FRET. *Anal Chem* **91**, 14936-14942, (2019).
